# Seizure pathways: A model-based investigation

**DOI:** 10.1371/journal.pcbi.1006403

**Published:** 2018-10-11

**Authors:** Philippa J. Karoly, Levin Kuhlmann, Daniel Soudry, David B. Grayden, Mark J. Cook, Dean R. Freestone

**Affiliations:** 1 Department of Biomedical Engineering, The University of Melbourne, Parkville, Australia; 2 Department of Medicine, St Vincent’s Hospital, The University of Melbourne, Fitzroy, Australia; 3 Brain Dynamics Lab, Swinburne University of Technology, Hawthorn, Australia; 4 Centre for Neural Engineering, The University of Melbourne, Parkville, Australia; 5 Department of Electrical Engineering, Technion, Haifa, Israel; 6 Seer Medical Pty, Melbourne, Australia; Ghent University, BELGIUM

## Abstract

We present the results of a model inversion algorithm for electrocorticography (ECoG) data recorded during epileptic seizures. The states and parameters of neural mass models were tracked during a total of over 3000 seizures from twelve patients with focal epilepsy. These models provide an estimate of the effective connectivity within intracortical circuits over the time course of seizures. Observing the dynamics of effective connectivity provides insight into mechanisms of seizures. Estimation of patients seizure dynamics revealed: 1) a highly stereotyped pattern of evolution for each patient, 2) distinct sub-groups of onset mechanisms amongst patients, and 3) different offset mechanisms for long and short seizures. Stereotypical dynamics suggest that, once initiated, seizures follow a deterministic path through the parameter space of a neural model. Furthermore, distinct sub-populations of patients were identified based on characteristic motifs in the dynamics at seizure onset. There were also distinct patterns between long and short duration seizures that were related to seizure offset. Understanding how these different patterns of seizure evolution arise may provide new insights into brain function and guide treatment for epilepsy, since specific therapies may have preferential effects on the various parameters that could potentially be individualized. Methods that unite computational models with data provide a powerful means to generate testable hypotheses for further experimental research. This work provides a demonstration that the hidden connectivity parameters of a neural mass model can be dynamically inferred from data. Our results underscore the power of theoretical models to inform epilepsy management. It is our hope that this work guides further efforts to apply computational models to clinical data.

## Author summary

A fundamental question in clinical neuroscience is how and why the brain generates epileptic seizures. To address this problem it is important to unify theoretical models of seizure mechanisms with clinical data. This study investigated a large database of human epileptic seizure recordings. Model inversion was used to track seizure dynamics through the lens of a mathematical model for cortical regions. These models can reveal the relative activity and coupling between excitatory, inhibitory and pyramidal neural populations that cannot be directly measured. Measuring cortical dynamics during seizures can provide insight into epilepsy, and facilitate new treatment strategies. Our analysis of connection strengths revealed important aspects of seizure onset and seizure termination. Our findings have implications for understanding seizure mechanisms and treating epilepsy.

## Introduction

Understanding how and why the brain generates spontaneous seizures is an unsolved problem in neuroscience. The medical implications of seizures are profound, with over 50 million people affected by epilepsy, and at least 30% not adequately controlled by available therapies [1]. Surgical treatment does not provide complete seizure freedom for all patients [2], and novel drugs have not greatly improved on the level of seizure freedom that can be achieved [3]. On the other hand, data-driven, computational techniques have shown early promise in obtaining a more individualized picture of a patient’s seizures, which may shed new light on mechanisms of seizures and lead to targeted treatment strategies [4, 5].

Patient-specific, computational models can provide unique insight into seizure mechanisms, and are well accepted in the study of epilepsy [6]. In particular, lumped parameter neural mass models [7, 8] have been extensively used to investigate cortical activity during epileptic seizures [9–11]. These models describe seizures as state transitions in the brain [12] that arise from endogenous noise perturbations or ‘pathways through the parameter space’ of a neural model [13]. Clinically, it is recognized that electrographic (EEG) recordings of seizures show stereotypical changes in the signal morphology that are regarded as state changes (i.e. between interictal, peri-ictal, and ictal states) [14]. Despite the ubiquity of neural mass models to study seizure transitions, the translation of these theoretical insights into clinical practice has not been widely realized.

The validation of neural network models to aid clinical decision making has made some advances in diagnosis [15], and surgical planning [16–19]. Ideally model-based techniques can also improve outcomes at earlier stage interventions, such as drug selection. Another area models can aid treatment may be in seizure forecasting [20, 21], or the design of electrical counter-stimulation (using model predictive control) [22–24]. A fundamental hurdle to overcome is validating theoretical models of brain dynamics in a clinical setting. This hurdle largely exists due to the difficulty of obtaining *in vivo* neural recordings from humans. Whilst simulation has proven valuable to generate new hypotheses regarding the mechanisms of seizures, a complete validation must unite empirical data with theoretical models and demonstrate that models have predictive value, as well as being descriptive of the data [4, 5].

Model inversion is a powerful approach to combine patient-specific recordings with accepted principles of brain structure and function encapsulated by the parameters of a neural model [25]. Previous work has outlined a generalizable framework to estimate the most likely states and parameters of a neural model given observed data [25]. For many years this problem was intractable for non-linear neural models [26]. Previously, model inversion has relied upon simplifying assumptions, such as linearization, or sampling techniques [27]. Another approach is to re-frame the problem, so that the objective is to find the most likely model to generate the observed data. In this way, the estimation is conditioned on the model space, which is generally explored via some heuristic model selection criteria [28, 29]. Alternatively, the inversion can be conditioned on the data, where the most likely model is identified using an assumed density (Kalman) filter [30]. This approach has been validated for investigation of seizure dynamics [31–33]. Recent advances have also incorporated a fast, semi-analytic solution to handle the propagation of estimates through the non-linear neural mass equations [25, 34, 35]. Model inversion techniques that enable time-varying estimates of key parameters provide a powerful means of inferring cortical mechanisms from functional neuroimaging data. This is particularly true for EEG/ECoG data, which has high temporal resolution. The ability to update estimates with each new data point can lead to insights into ictal dynamics that evolve over fast time scales.

Statistical observations from data are also important to validate models of seizure transitions. Some studies have investigated the distributions of times spent in different seizure states [36]. Models that are predictive of higher-order statistics derived from many seizures are more convincing than models which are only descriptive of (or fitted to) individual seizures [4]. An important observation is that the distribution of times spent in the ictal state has a patient-specific peak [37], rather than a uniform distribution. These peaks indicate that patients have a characteristic seizure duration, or trajectory length. Intriguingly, a subset of patients showed a distinctly bimodal distribution of seizure durations, indicating two populations of seizures (long and short) [37]. We hypothesize that these distributions reveal a crucial aspect of seizure dynamics, which should not be neglected in computational modeling.

## Materials and methods

This work presents a large-scale, model-based investigation to address the question of how multiple (long and short) seizure trajectories arise in the brain. Model inversion was performed for the largest database of human seizures recorded in individual patients [38]. Using this database, we have previously demonstrated that there are two populations of seizure duration [37]. The current study investigated different seizure pathways and mechanisms through the lens of a neural mass model (using the formulation of Jansen and Rit, 1995 [8]). The following sections outline the data, model and estimation techniques. Further detail is provided in S1 Appendix, and code is available online (https://github.com/pkaroly/Data-Driven-Estimation).

### Seizure data

Seizure mechanisms were investigated for continuously recorded ECoG from 12 patients with focal epilepsy monitored during a previous clinical trial [38]. All subjects were implanted with intracranial electrode arrays with a total of 16 platinum iridium contacts around the seizure onset zone. The ECoG was sampled at 400 Hz and wirelessly relayed to an external, portable personal advisory device. Seizure detection was automated and reviewed by expert clinicians. This study used data from 3010 clinical seizures (average 250 per patient). Seizures were either associated with confirmed clinical symptoms or were electrographically similar to clinical seizures. Other epileptiform discharges without clinical symptoms were excluded. All seizures had onset and offset labelled by expert epileptologists. For further details on the data collection procedures the reader is referred to Cook et al. (2013) [38].

A similar procedure to that outlined by Cook et al. (2016) [37] was used to identify patients with bimodal seizure durations. Both k-means clustering and Gaussian mixture model fitting were used to test for bimodality. Clusters were assigned for one, two and three seizure populations (based on the logarithm of seizure duration in seconds). The optimal number of clusters was determined using gap criteria [39].

The current study used patients who had at least 20 seizures that had a lead time of one hour. Recordings were used from five minutes before seizure onset, until one minute after seizure offset. Seizures with telemetry dropouts were excluded from analysis. Data were bandpass filtered (second-order, zero-phase Butterworth filter) from 1 Hz to 180 Hz with a notch filter at 50 Hz (second-order, zero-phase Butterworth filter). The energy of the signal was computed for a 1s sliding window (50% overlap) as $\text{energy}=\sum_{n=1}^{N}x^{2}$.

### Neural model

The states (mean membrane potentials) and parameters (synaptic connectivity strength) of neural mass models were fitted to data recorded during epileptic seizures. The formulation of the neural mass model in the following section is derived from the model introduced by Jansen and Rit (1995) [8]), and has also been outlined in our previous work [33, 34]. The neural mass model is suitable to model ECoG measured at this scale (electrodes approximately 5mm in diameter with spacing on the order of centimeters), in line with similar neural models used to describe EEG/MEG activity [10, 11, 40]. A single, independent neural model was fitted to each ECoG channel (16 models in total). Neural models were not coupled between channels; hence, estimates primarily captured local connection strengths within a single cortical region. The input parameter, *u* described non-local inputs to the pyramidal population.

**Fig 1:**
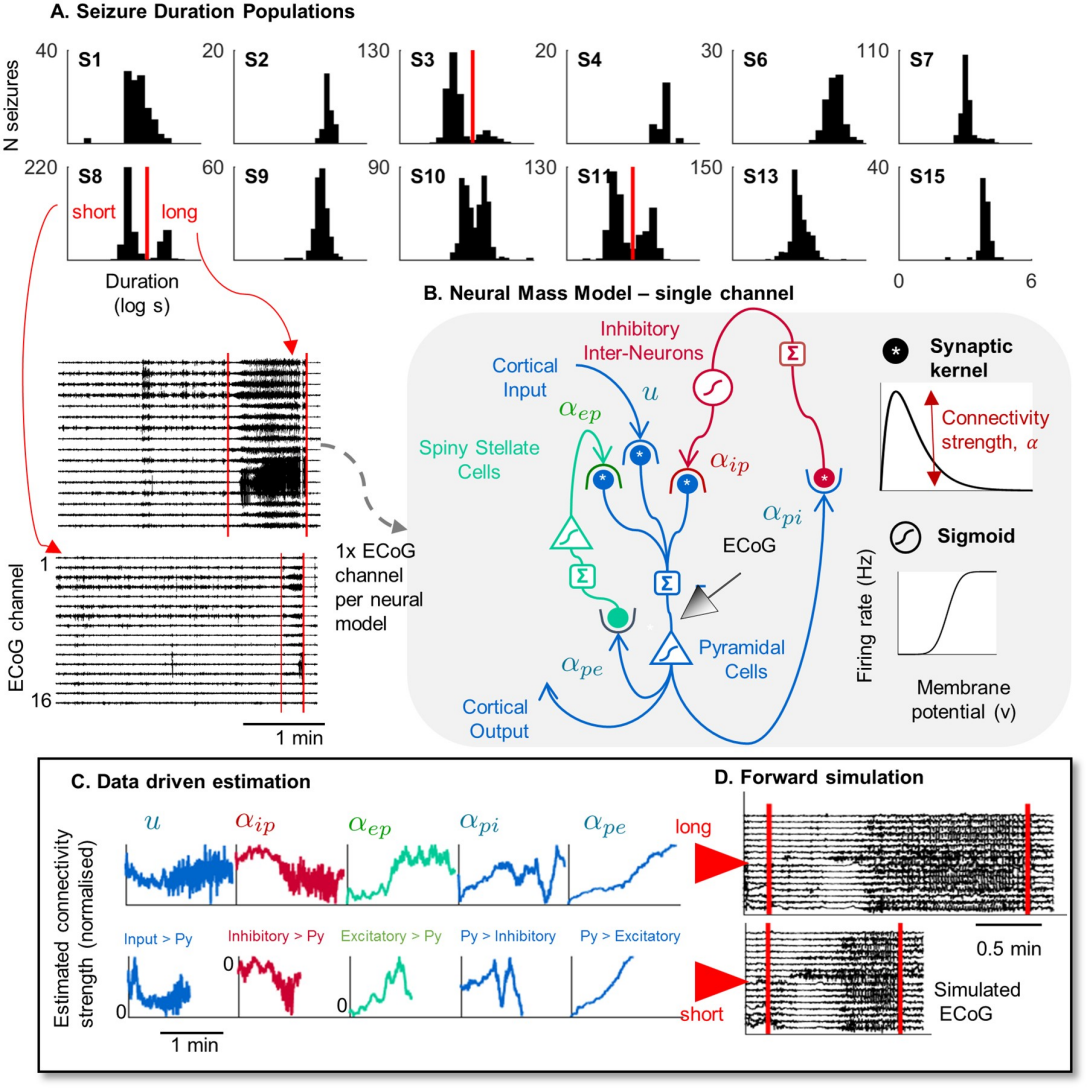
Data driven estimation using a neural mass model. **A**. Histograms of seizure durations (log seconds) for the twelve patients considered in this study, with examples of ECoG during a long and short seizure from a single patient, S8. **B**. Schematic of the neural model. Each synapse is defined by a convolution kernel (converting pre-synaptic firing rates to post-synaptic membrane potentials). Each population is defined by a sigmoid function (converting mean membrane potential to average firing rate). **C**. Example estimation time series of the five connectivity parameters during a long and short seizure for one patient. **D**. Deterministic forward simulation using the estimated parameters in C (red lines mark actual seizure onset and offset).

The Jansen and Rit model consists of three neural populations (excitatory, inhibitory and pyramidal). Neural populations were described by their time varying mean membrane potential, *v*_*n*_, which is the sum of contributing mean post-synaptic potentials, *v*_*mn*_ (post-synaptic and pre-synaptic neural populations are indexed by *n* and *m*, respectively). For the current model, the index *n* (post-synaptic) represents either pyramidal (*p*), excitatory (*e*) or inhibitory (*i*) populations, as shown in Fig 1.

The post-synaptic potential, *v*_*mn*_ arises from the convolution of the input firing rate, *ϕ*(*v*_*n*_), with the post-synaptic response kernel,

$$\begin{array}{c} v_{mn}\left(t\right)=\alpha_{mn}\int_{-\infty }^{t}h_{mn}\left(t-t^{\prime }\right)\phi (v_{n}\left(t^{\prime }\right))\;dt^{\prime }, \end{array}$$ (1)

where *α*_*mn*_, which are the estimation parameters, represent lumped connectivities that incorporate average synaptic gain, number of connections, and average maximum firing rate of the presynaptic populations. *ϕ*(*v*_*n*_) is the sigmoid function

$$\begin{array}{c} \phi (v)=\frac{1}{2}(\text{erf}(\frac{v-v_{0}}{\varsigma })+1) \end{array}$$ (2)

where *v*_0_ = 6mV, and *ς* = 0.0030 (as defined by Freestone et. al. (2014) [25]).

The convolution in Eq 1 can be written as two coupled, first-order, ordinary differential equations,

$$\begin{array}{c} \frac{dv_{mn}}{dt}=z_{mn}\\ \frac{dz_{mn}}{dt}=\frac{\alpha_{mn}}{\tau_{mn}}\phi_{mn}-\frac{2}{\tau_{mn}}z_{mn}-\frac{1}{\tau_{mn}^{2}}v_{mn}. \end{array}$$ (3)

where *τ*_*mn*_ is a lumped time constant. The values of *τ*_*ep*_, *τ*_*pe*_, and *τ*_*pi*_ were fixed to 10ms and the value of *τ*_*ip*_ to 20ms, as defined by Jansen & Rit (1995) [8].

External (non-local) inputs to the pyramidal population are modeled as an additive term affecting the pyramidal membrane potential,

$$\begin{array}{c} v_{p}\left(t\right)=v_{pe}\left(t\right)-v_{pi}\left(t\right)+u\left(t\right). \end{array}$$ (4)

The recorded ECoG for each channel, *i*, is derived from the average pyramidal membrane potential of each independent neural mass model (resulting 16 disconnected models in the estimation),

$$\begin{array}{c} y^{i}\left(t\right)=v_{p}^{i}\left(t\right) \end{array}$$ (5)

The neural model can be expressed in matrix notation

$$\begin{array}{c} \dot{x}\left(t\right)=Ax\left(t\right)+B\vec{\phi }(Cx(t)), \end{array}$$ (6)

where $x\in R^{N_{x}}$ is a state vector representing the postsynaptic membrane potentials generated by each population synapse and their time derivatives. There are two states per synapse and *N*_*x*_ = 2*N*_*s*_ is the total number of states, where, for *N*_*s*_ synaptic connections in the models, the state vector is of the form,

$$\begin{array}{c} x={\left[\begin{array}{ccccc} v_{1} & z_{1} & \ldots & v_{N_{s}} & z_{N_{s}} \end{array}\right]}^{⊤}. \end{array}$$

The definitions of **A**, **B**, and **C** are provided in S1 Appendix.

The observation equation is of the form

$$\begin{array}{c} y(t)=Hx(t)+v(t), \end{array}$$ (7)

where $H\in R^{N_{x}\times N_{y}}$ is the observation matrix, $v\left(t\right)∼N(0,R)\in R^{N_{y}}$ is the observation noise, and *N*_*y*_ is the number of observations (here *N*_*y*_ = 1 as each neural mass model describes a single ECoG channel). As our measurement function is linear, **H** is simply an index vector of zeros and ones that defines the average pyramidal membrane potential given by Eq 4.

### State and parameter estimation

A joint state (membrane potentials) and parameter (external input and connectivity strengths) estimation algorithm was implemented for every sample of the recorded ECoG. To obtain estimates it was necessary to augment the state-space representation of the neural model. To define the augmented model, we first define a vector of parameters as

$$\begin{array}{c} \theta ={\left[\begin{array}{ccccc} u & \alpha_{pe} & \alpha_{pi} & \alpha_{ip} & \alpha_{ep} \end{array}\right]}^{⊤}. \end{array}$$

The dynamics for the parameter are modeled as a random walk

$$\begin{array}{c} \dot{\theta }=0. \end{array}$$ (8)

The state vector **x** and the parameter vector *θ* are concatenated to form the augmented state vector,

$$\begin{array}{c} \xi ={\left[\begin{array}{cc} x^{T} & \theta^{T} \end{array}\right]}^{⊤}. \end{array}$$ (9)

Our augmented state-space model is

$$\begin{array}{c} \xi_{t}=A_{\theta }\xi_{t-1}+B_{\theta }\phi \left(C_{\theta }\xi_{t-1}\right)+w_{t-1}, \end{array}$$ (10)

where $w_{t}∼N(0,Q)$. The state vector $\xi \in R^{N_{\xi }\times 1}$ and matrices **A**_*θ*_, **B**_*θ*_, and **C**_*θ*_ are $\in R^{N_{\xi }\times N_{\xi }}$ and have the form

$$\begin{array}{c} A_{\theta }=[\begin{array}{cc} A & 0\\ 0 & I \end{array}],B_{\theta }=[\begin{array}{cc} B & 0\\ 0 & 0 \end{array}],C_{\theta }=[\begin{array}{cc} C & 0\\ 0 & 0 \end{array}]. \end{array}$$ (11)

For simplicity we will drop the subscript *θ* on the system matrices, as the remainder of the equations refer to the augmented model.

The estimation scheme uses an assumed density filter. This filter provides the minimum mean squared error estimates for the states and parameters, under the assumption that the underlying probability distribution is Gaussian (the assumed density). Formally stated, the aim of estimation is to compute the most likely posterior distribution conditioned on previous measurements,

$$\begin{array}{c} {\hat{\xi }}_{t}^{+}=E[\xi_{t}|y_{1},y_{2},\ldots ,y_{t}] \end{array}$$ (12)

$$\begin{array}{c} {\hat{P}}_{t}^{+}=E[\left(\xi_{t}-{\hat{\xi }}_{t}^{+}\right){\left(\xi_{t}-{\hat{\xi }}_{t}^{+}\right)}^{⊤}], \end{array}$$ (13)

The estimator proceeds in two stages; prediction and update. In prediction, the prior distribution (obtained from the previous estimate) is propagated though the neural mass equations. This step provides the so called *a priori* estimate, which is a Gaussian distribution with mean and covariance,

$$\begin{array}{c} {\hat{\xi }}_{t}^{-}=E[\xi_{t-1}|y_{1},y_{2},\ldots ,y_{t-1}] \end{array}$$ (14)

$$\begin{array}{c} {\hat{P}}_{t}^{-}=E[\left(\xi_{t-1}-{\hat{\xi }}_{t-1}^{+}\right){\left(\xi_{t-1}-{\hat{\xi }}_{t-1}^{+}\right)}^{⊤}]. \end{array}$$ (15)

In the second stage, a Bayesian update is performed to shift the estimated posterior based on the observed data, giving the *a posteriori* distribution,

$$\begin{array}{cc} {\hat{\xi }}_{t}^{+}= & {\hat{\xi }}_{t}^{-}+K_{t}\underset{\text{ECoG}\;\text{prediction}\;\text{error}}{\underset{︸}{\left(y_{t}-H{\hat{\xi }}_{t}^{-}\right)}}.\\ {\hat{P}}_{t}^{+}= & \left(I-K_{t}H\right){\hat{P}}_{t}^{-}, \end{array}$$ (16)

where K is the Kalman gain (readers are referred to [27] for a detailed description of the Kalman filter). After each time step, the *a posteriori* estimate becomes the prior distribution for the next time step, and the filter proceeds.

In general, the Kalman filter equations do not have a solution for nonlinear model or measurement functions. Previous efforts to use Kalman filtering on the nonlinear neural mass model have relied on simplifying assumptions (either linearization of the model, or sampling to estimate the posterior distribution). This work applied an exact, semi-analytic solution for the mean and covariance of a multivariate Gaussian distribution transformed by the nonlinear neural mass model. This solution provides the *a priori* estimate of the mean and covariance (see S1 Appendix for details).

As the observation function is linear, the updated (*a posteriori*) mean and covariance are obtained trivially using Eq 16.

The Kalman filter requires ${\hat{\xi }}_{0}^{+}$ and ${\hat{P}}_{0}^{+}$ to be initialized to provide the *a posteriori* state estimate and state estimate covariance for time *t* = 0. The other parameters that must be initialized are the model and measurement noise, **Q** and **R**, respectively. Further details of filter initialization are given in the S1 Appendix.

## Results

Fig 1A shows an overview of the estimation scheme. The following sections present data from twelve patients with focal epilepsy. The data consist of over 3000 clinical seizures (average 250 seizures per patient). Of these twelve patients, three showed bimodal distributions of seizure durations (Patient 3, 8 and 11). Note that all patients showed that either one or two clusters were optimal for seizure durations, and for all patients, k-means and Gaussian mixture modelling aligned on the same optimal number of clusters.

### Estimated connectivity within neural models describe seizure transitions

An assumed density filter was used to track the time-varying states and parameters of neural mass models during every seizure (as seen in Fig 1). This estimation technique finds the most likely model given the observed ECoG data. Importantly, the model is updated at every time step, so there is no loss of temporal resolution. Estimated states were mean membrane potentials, and parameters (alpha parameters in Fig 1B and 1C), which were the external input and average synaptic strengths between pyramidal, non-pyramidal excitatory, and inhibitory neural populations. In this way, the neural models provided an estimate of the average activity and effective connectivity within intracortical circuits [25]. We found that slow changes in the synaptic connectivity parameters led to seizure transitions in the neural models. As seen in Fig 1C, a deterministic forward simulation of neural models using time-varying connectivity estimates reproduced the beginning and end of seizures.

It is important to also quantify estimation accuracy before proceeding. A full summary of the model and estimation errors is given in the Supplementary Material. However, it is worth noting briefly that errors between the estimated signal and real data were small (see S1 Fig). The mean squared error ranged from 0.2-0.9 mV when averaged across all seizures (note that the mean amplitude of the measured ECoG signal ranges from approximately 25-100mV). The uncertainty (covariance) of state variables and parameters was also small (see S2 and S3 Figs), suggesting that key seizure activity was well described by the model, rather than by the residuals. Across patients, the mean covariance ranged from 2-16% for state variables, and from 0.1-10% for connectivity parameters (expressed as a percentage of the estimated value). Numerical instability of the filter was occasionally observed (for all patients, instability occurred for less than 1% of the data). Estimates that became unstable were removed from further analysis.

### Long and short seizures show distinct signal evolution

**Fig 2:**
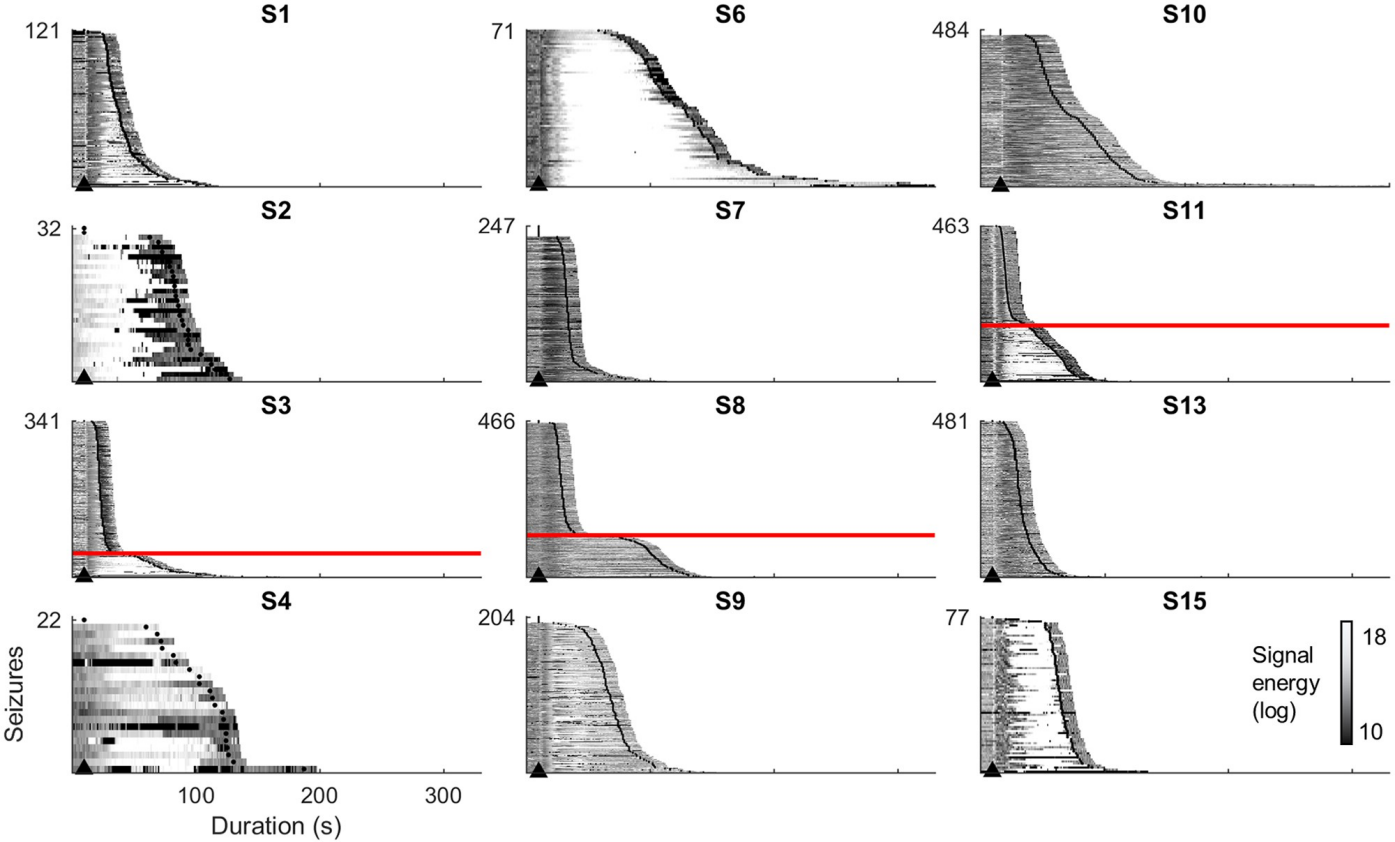
Seizure energy. Average signal energy during evolution of seizures, sorted by duration, from 10s before seizure onset (marked by arrowhead) to 10s after seizure termination (according to clinicians’ marking). Red lines mark the divisions between long and short populations of seizures within bimodal subjects. Energy for individual electrode channels is provided in the supplementary material.

Fig 2 shows the average energy of recorded ECoG during every seizure (averaged across 16 electrode channels). Patients’ seizures showed strikingly consistent patterns of signal energy between seizures. These patterns were generally time locked to seizure onset, as demonstrated by the vertical alignment of energy changes (note that Patients 2, and 4 did not show a vertically aligned onset pattern). Long and short seizures began similarly, but evolved differently (see Patients 3, 8, and 11). Long seizures entered a secondary phase where energy increased. Short seizures and the early phases of long seizures were characterized by an energy reduction (note the darker vertical band following seizure onset). Some patients’ seizures only showed the “long” stereotypical pattern with a high-energy phase (see Patients 2, 4, 6, 9, and 15). Patient 13 had only low energy, stereotypical “short” seizures. Patient 7 had a large majority of short seizures, with a small number evolving to have increased energy.

These two patterns of seizure energy suggest that long and short seizures reflect distinct event types, each with a characteristic electrographic evolution. We hypothesized that these stereotypical signal patterns represent two alternative seizure trajectories, which could be differentiated by their onset and/or offset mechanisms. Note that although the average energy (averaged across electrode channels) was presented, the observed patterns were consistent across different channels. Full plots are provided in the Supplementary Material (S8 to S19 Figs).

### Seizure trajectories

**Fig 3:**
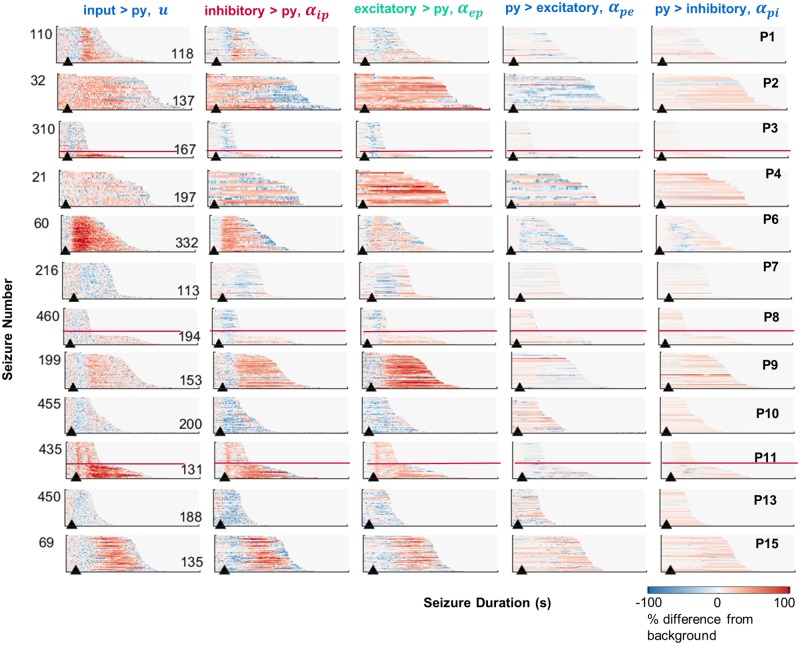
Estimated changes in parameter trajectories during every seizure. Each subpanel represents the connectivity strength for all seizures (sorted by duration) of a single representative channel for each patient (P1: Ch3, P2: Ch1, P3: Ch2, P4: Ch2, P6: 7, P7: Ch15, P8: Ch11, P9: Ch4, P10: Ch13, P11: Ch9, P13: Ch5, P15: Ch1). Data for all channels is provided online (https://github.com/pkaroly/Data-Driven-Estimation/tree/master/figures/connectivity). Parameters are expressed as a percentage change from their pre-ictal background values (where zero reflects no change from the pre-ictal period). The pre-ictal period was defined from two minutes to one minute before seizure onset. Red lines mark the divisions between long and short populations of seizures within bimodal subjects.

Fig 3 presents the dynamic estimation results for the five connectivity parameters of a neural model during every seizure. Seizures followed a remarkably consistent trajectory through the parameter space of the neural mass models, showing similar patterns across all events for an individual. This indicates that seizure transitions follow a stereotypical pathway. Note that transitions during the seizure are locked to the onset time (as demonstrated by the vertical banding in parameter changes). A higher contrast (normalized) version of these patterns is provided in S4 Fig, to more clearly expose connectivity patterns.

For all patients, the strongest ictal changes in connectivity strength occurred for in-going connections to the pyramidal neurons (the first three columns of Fig 3). Conversely, outgoing pyramidal connections (to inhibitory and excitatory neurons) were more stable over the durations of the seizures, demonstrated by values which were closer to zero (reflecting no change from baseline), and less vertical patterning (reflecting no stereotypical transitions during seizures). Patient 6 was one possible exception, showing some decrease in outgoing pyramidal connections (6D and 6E).

Note that although neural models were fitted independently to all 16 electrode channels, Fig 3 shows results for a single example channel per patient. The data associated with every channel generates 60 full page figures, which are provided in an online repository (https://github.com/pkaroly/Data-Driven-Estimation). The consistency of stereotypical patterns between channels is investigated in the following sections.

**Fig 4:**
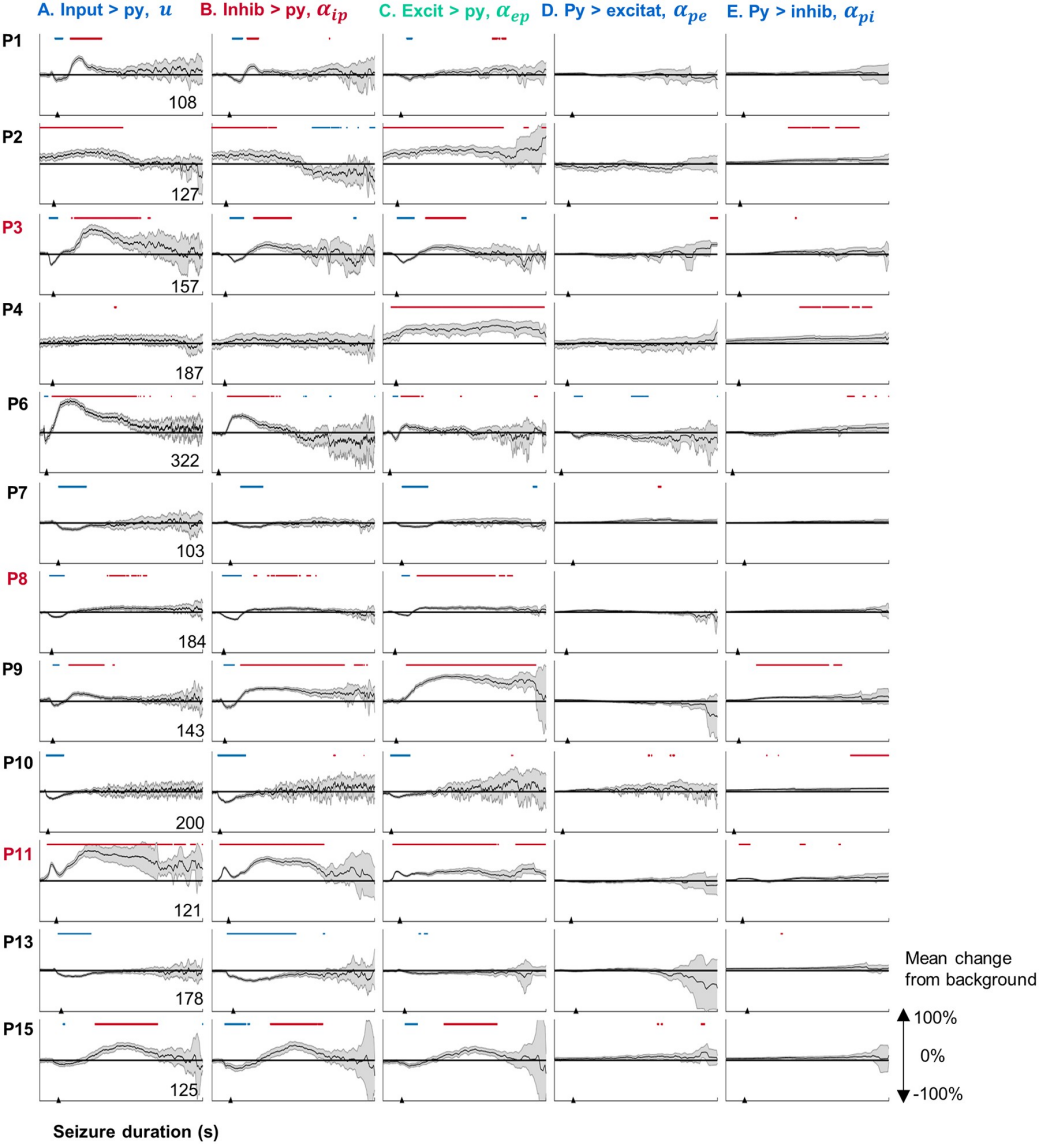
Average changes in parameter trajectories. Each subpanel represents the mean connectivity strength (averaged across seizures) for each patient (grey shading represents the 95% confidence bounds of the mean). For each patient a single representative channel was chosen (P1: Ch3, P2: Ch1, P3: Ch2, P4: Ch2, P6: 7, P7: Ch15, P8: Ch11, P9: Ch4, P10: Ch13, P11: Ch9, P13: Ch5, P15: Ch1). Data for all channels is provided online (https://github.com/pkaroly/Data-Driven-Estimation/tree/master/figures/connectivity). Parameters are expressed as a percentage change from their pre-ictal background values (where zero reflects no change from the pre-ictal period). The pre-ictal period was defined from two minutes to one minute before seizure onset. Significant (p < 0.05) changes are marked in blue (decrease from baseline) and red (increase from baseline) at the top of each plot. Bimodal patients are shown with red labels. Columns from A-E show the connectivity parameters: A) external input to pyramidal neurons, B) inhibitory to pyramidal connectivity, C) excitatory to pyramidal connectivity, D) pyramidal to excitatory connectiviy, E) pyramidal to inhibitory connectiviy.

Fig 4 shows the mean change in connectivity strength during seizures. A consistent motif was a decrease followed by an increase in ingoing connections to the pyramidal population (see columns A and C for Patients 1, 3, 6, 8, 9, and 15). Patient 11 showed the same motif, but with connectivity strength always above baseline. Patients 7, 10, and 13 showed only decreases in ingoing pyramidal connections. Patients 2 and 4 demonstrated only strengthened connections.

There were three classes of ictal parameter transitions: decrease, increase, and decrease-then-increase, where connections into the pyramidal populations were on average weaker, stronger, or weakened then strengthened (compared to a pre-ictal baseline), respectively. These classes aligned well with the stereotypical seizure evolution patterns that were identified based on signal energy (Fig 2). For instance, “decrease-then-increase” patients (1, 3, 6, 8, 9, and 15) showed long seizures that began with lower energy and evolved into a higher energy state. The “increase” patients (2 and 4) showed primarily high energy seizures without an obvious alignment to seizure onset. The “decrease” patients (7, 10, and 13) showed short, low-energy seizures.

Outgoing connections from pyramidal cells to excitatory/inhibitory populations showed little to no change. For some patients (2, 4, 9 and 10), a slight increase in pyramidal to inhibitory strength was observed.

**Fig 5:**
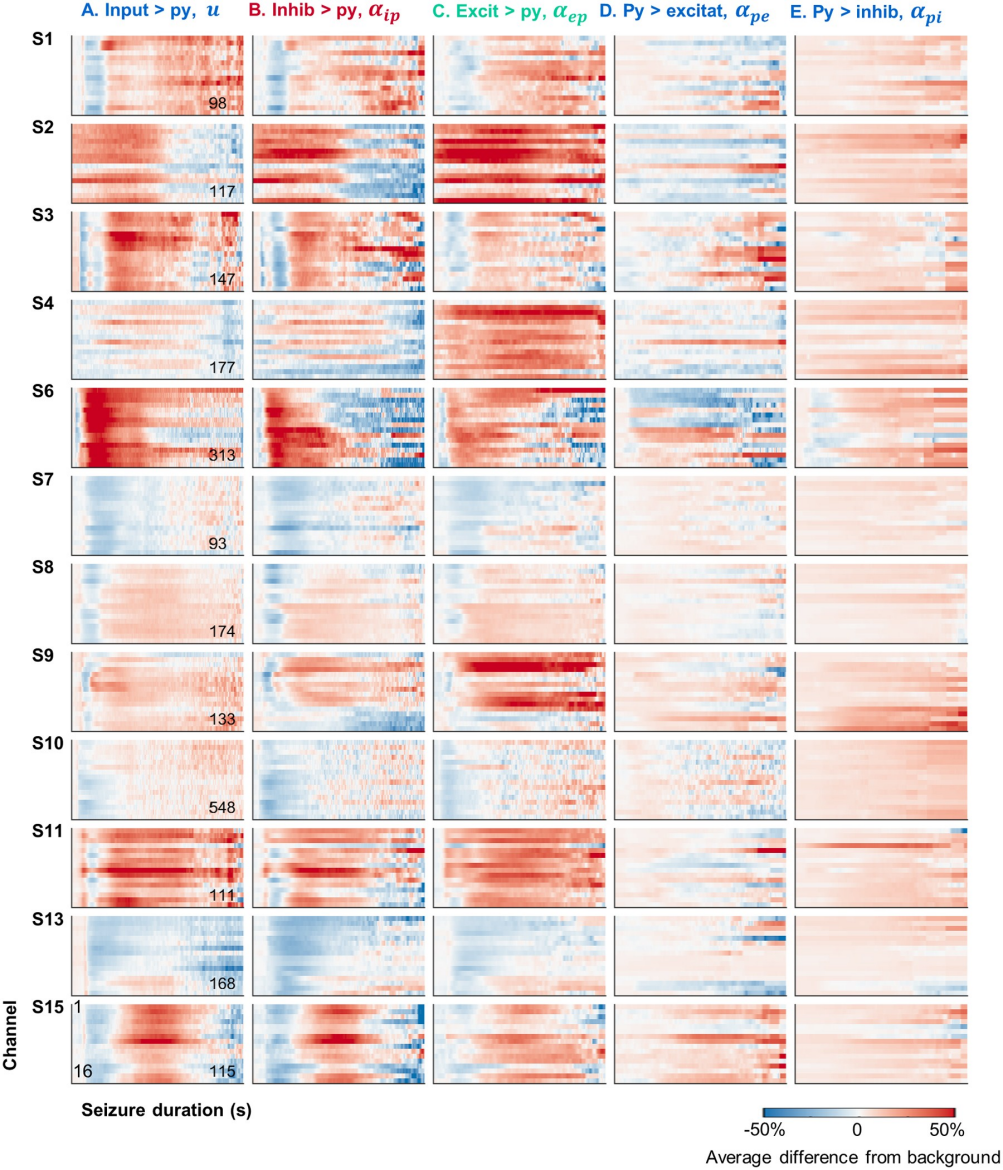
Inter-channel changes in parameter trajectories. Each subpanel represents the mean connectivity strength (averaged across seizures) for all channels (y-axis) for each patient. Parameters are expressed as a percentage change from their pre-ictal background values (where zero reflects no change from the pre-ictal period). The pre-ictal period was defined from two minutes to one minute before seizure onset. Significant changes are shown in the Supplementary Material (S6 Fig). Columns from A-E show the connectivity parameters: A) external input to pyramidal neurons, B) inhibitory to pyramidal connectivity, C) excitatory to pyramidal connectivity, D) pyramidal to excitatory connectiviy, E) pyramidal to inhibitory connectiviy.

Fig 5 shows the average seizure trajectory for all channels. Trajectories were qualitatively similar across channels. Most subjects showed focal patterns in which a subset of channels demonstrated connectivity changes during seizures while other channels did not have significantly increased or decreased connectivity during seizures (compared to a pre-ictal baseline period). Such patterns were not surprising, given that all subjects had focal seizures, which typically appear first on a subset of EEG channels before spreading. Apart from focal connectivity changes, there was some inter-channel variability at the ends of seizures. Subject 6 showed some channels with increased connection strengths and others with decreased strengths. Subject 9 showed increased inhibitory connections across most channels but decreased inhibition on a subset of channels (Fig 5, subpanel 9B), that occurred toward the ends of seizures. Overall, significant changes in connectivity (above or below baseline) followed the same stereotypical, patient-specific pattern across all channels. Exceptions to this consistency were observed for a few subjects in the later stage of seizures (significant changes are shown in Supplementary Material S6 Fig). In other words, there were no channels with markedly different trajectories; changes in connectivity were either in the same direction or showed no significant change from baseline. This consistency supports the finding of characteristic pathways of epileptic seizures, although these pathways were only observed on a subset of (possibly focal) channels, while other channels did not show altered connectivity patterns during seizures.

### Seizure transitions

Overall, there was no difference in the average connection strength trajectories for long compared with short seizures (when connections were averaged across seizures in the long and short populations, respectively; see S5 Fig for details). Therefore, we hypothesized that short and long seizures were primarily differentiated by termination (i.e. both types follow a similar path from onset, with short seizures terminating earlier). This hypothesis was tested by measuring the correlation between connection strength (now averaged across 16 electrode channels) and seizure duration before onset and offset (correlation results were qualitatively similar when evaluated for individual electrode channels, and are provided in S7 Fig).

**Fig 6:**
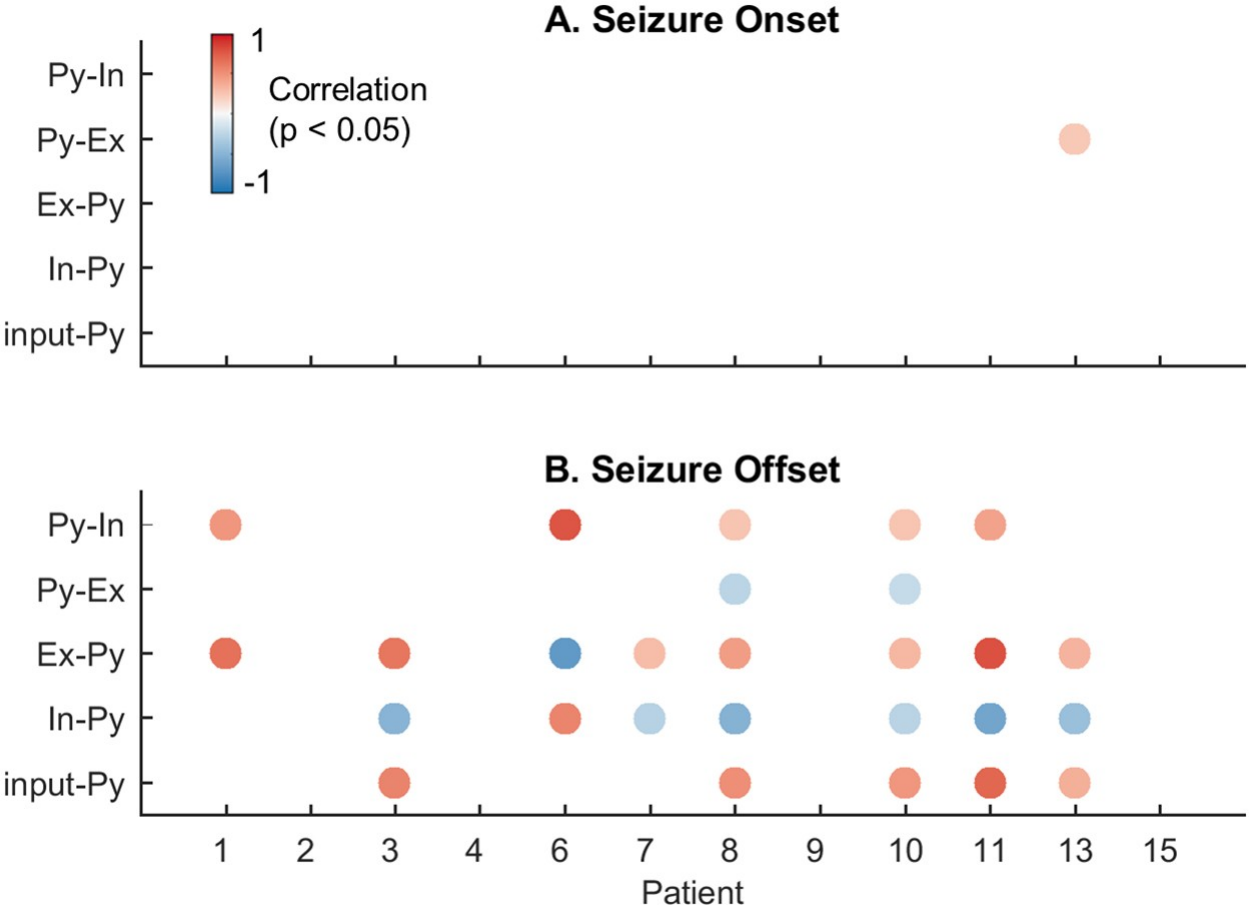
Correlation between connectivity and seizure duration. Correlation was measured between seizure duration and average connectivity strength (averaged across electrode channels) taken 5s before seizure onset (Panel A), and 5s before seizure offset (Panel B). Only significant correlation values are shown (*p* < 0.05). A Bonferroni correction for multiple comparisons was performed before computing significance, where the 0.05 significance level was divided by 60 (12 patients and 5 parameters).

Fig 6 shows that almost no patients showed significant correlation between seizure duration and onset dynamics. In other words, there was no relationship between average connection strength and seizure duration at the outset (measured over a 5s window prior to seizure onset). However, at 5s before seizure offset, there were strong correlations for all connections. In general, longer seizures were associated with increased excitatory inputs and decreased inhibition to the pyramidal cells. Bimodal patients (3, 8, 10, and 11) all showed a similar relationship between connectivity strength and seizure duration. Four other patients also showed significant correlations; therefore, correlations do not arise purely because of the two duration populations.

## Discussion

Analysis of seizure energy (Fig 2) suggests there are two broad categories of focal seizures: short (low energy) and long (high energy) seizures. Estimation of patients’ seizure trajectories through the parameter space of a neural model revealed characteristic mechanisms underlying these different energy states (Fig 3). During the low energy phase of seizures, estimation showed decreased connectivity strength of ingoing connections to pyramidal cells. Seizures with high energy showed increased connection strength. This pattern was maintained as seizures evolved through time, with several patients showing a motif of decreased then increased connection strengths, corresponding to low energy seizures that evolved into a high-energy state.

Based on their characteristic seizure durations three patient subtypes were defined; those with exclusively short, exclusively long, or bimodal populations of seizure types. Understanding how these different patterns of seizure evolution arise may provide new insights into brain function, and guide treatment for epilepsy, as specific therapies may have preferential effects on the various parameters that could potentially be individualized. This study showed that long and short seizures reflect different underlying mechanisms in a neural model. Mechanistic differences arose almost exclusively before seizure offset, and were not evident prior to onset (Fig 6). Therefore, we conclude that seizures follow the same trajectory until termination. Apart from a bimodal distinction, connectivity patterns were strikingly similar during the evolution of each patient’s seizures; although highly patient-specific. This suggests that, once initiated, seizures follow an individualized and deterministic path through the parameter space of a neural model. It is remarkable to see these parametric pathways maintained across hundreds of seizures (see Fig 3), and over many years recording duration.

### Seizure mechanisms

Patients were classified into three groups of connectivity patterns during seizures (seen in Fig 4): increased, decreased, and decreased-then-increased strength of ingoing connections to pyramidal cells. These parameter shifts may relate to distinct mechanisms of seizure onset. For the decrease, and decrease-then-increase patterns we speculate that seizures arise from either under-regulation or disinhibition of pyramidal neurons. The corresponding rebound of connection strength (in the decrease-then-increase group) may be linked to a regulatory mechanism that was not triggered for patients with shorter seizures (in the decrease group). Previous work using the neural mass model confirms that inhibitory populations are likely to play a role in generating epileptiform activity, with the time scale of inhibitory dynamics also highly relevant [10]. There were also two patients who showed only increased connection strength to pyramidal cells (in Fig 4, Patient 2 showed all connections were increased and Patient 4 showed an increase of excitatory inputs). For these patients, seizures may have been driven by over-excitation of pyramidal neurons.

There is a lack of consensus as to whether noisy fluctuations (multi-stability) or deterministic parameter changes (bifurcations) drive seizure onset/offset [4]. Other mechanisms, such as intermittency, may also be involved in seizure transitions [41, 42]. This study demonstrated that the transitions of connectivity parameters were locked to the onset of seizures, and not the offset (i.e. the patterns in Figs 3 and 2 arise when the seizures are aligned by start time, rather than end time). This finding suggests that there is a deterministic process conditioned on the start time of the seizure, whereas the lead up to seizure offset showed more stochasticity. Based on these results we speculate that seizure onset is more likely to occur through a deterministic process (as in a bifurcation), where the brain state is driven across some ‘point of no return’. Offset is more likely to result from noisy fluctuations. Other studies have hypothesized that seizures terminate as the result of a bifurcation [43, 44]. However, the brain’s state during a seizure may merely approach a critical transition, without crossing over [45]. Therefore, it is possible to observe signs of critical slowing (as in (Kramer et al., 2012)) yet still have seizure termination driven by noise [4, 46, 47].

The presence of characteristic seizure durations should inform theoretical approaches to modeling seizure transitions. For instance, in a bistable regime, where noisy fluctuations drive the transition between a fixed point and oscillatory (‘seizure-like’) state, characteristic dwell times can emerge for the different states [4, 46, 47]. Dwell times provide one candidate mechanism for characteristic seizure durations. Bimodal populations in some patients suggest that the brain can support two distinct seizure trajectories (short and long). It has been shown experimentally that different durations of seizures may arise as the result of distinct onset stimuli [48]. Explanations for multiple seizure types can also be derived from computational models. For instance, different background stability properties in a cortical model can result in two distinct types of seizures [49]. Multiple seizure trajectories can also arise from different onset bifurcations [43]. Similarly, multiple offset bifurcations could terminate seizures earlier or later, giving two populations of duration. The results of this work suggest that long and short seizures arise from distinct mechanisms of seizure termination. This hypothesis is supported by a recent study from Payne et al. (2018), which found that long and short seizures were associated with different durations of post-ictal suppression [50].

### Clinical implications

Knowledge of parameter transitions within neural models can increase the information extracted from EEG, informing new hypotheses of seizure mechanisms and guiding clinical practice. There is some evidence to suggest that the clinical classification of a seizure is predictable soon after its onset [51], in other words, the evolution of a seizure may be somewhat predetermined. Our results support the existence of predictable seizure types, and provide additional metrics (based on the parameters of a neural model) that may extend our understanding of traditional seizure types. The consistency of neural model parameters over many seizures suggests that, for some patients, seizure trajectories are established via repetition. The notion of ‘learned epilepsy’ [52], is an interesting interpretation of epileptogenesis whereby the abnormal process is learning and spontaneously repeating a pathological sequence, rather than the sequence itself (all brains can support seizures). For some patients, successful treatment strategies may involve disrupting or even reversing memorization of the seizure, rather than addressing an underlying cause [52]. On the other hand, the current results (Fig 3) also showed that seizure pathways were highly patient-specific and not all subjects’ trajectories were conserved over time.

Neural mass models have the potential to highlight the relative contributions of excitatory versus inhibitory connections during seizures. This information can guide whether GABAergic or glutamatergic drugs are required. Previous studies using neural mass models have demonstrated alterations in the balance of excitation and inhibition estimated from data recorded during seizures [40, 53, 54]. The current estimation technique enables previous efforts to be extended to investigate a large number of seizures. Some patients showed decreased inhibition at seizure onset, whereas others demonstrated increased excitation (Fig 4), potentially warranting different therapies. Furthermore, patients with two duration populations may require different strategies to terminate their seizures. Knowing in advance when two adjunct therapies are needed is an important clinical insight that can provide crucial benefits to patients with drug refractory epilepsy. This study found that long seizures were correlated with lower inhibition and higher excitation (in one patient, the reverse was the case), which can guide electrical stimulation designed to precipitate early termination of seizures.

The presented model inversion technique and results have wide-ranging applications. The parameter estimates were consistent across many seizures. Until now, it has not been possible to show consistency of models of seizure transition in ECoG due to the limited availability of long-term seizure recordings. Results also generalized across patients. Although the cohort of 12 patients was not large, prior studies have restricted model inversion of seizures to only one or two patients [13, 55–58]. Another important aspect is that the techniques can be generalized across models. The estimation filter is not specially formulated for the Jansen and Rit model used in this study but can be generalized to any model that uses the basic matrix representation provided in the derivation (see Supplementary Materiall S1 Appendix). That is, the approach can be applied to any combination of coupled neural populations or indeed any network model that can be represented by a linear component and a non-linear sigmoidal (error function) coupling term.

### Limitations and future work

Data driven modeling may provide the opportunity to identify which drug could be helpful for different classes of seizure, as different mechanisms of anti-epileptic drug action may preferentially effect the various connectivity parameters, though further validation of model predictions is needed to translate estimation results to clinical practice. Levels of AEDs have been related to features of the EEG signal [59, 60]. Therefore, it may be possible to extend this relationship to predicting the mode of action of an AED from an individual’s EEG. The time scale of connectivity changes may also be highly relevant to suppressing epileptic activity [10]. Future work should extend estimation to include time constants and investigate the utility of the outlined neural parameters to detect and predict drug action.

This study provided estimates of independent neural circuits for each channel of ECoG. Previous studies using coupled neural mass models have highlighted the importance of inter-channel interactions, particularly for seizure propagation [61]. However, this work considered local coupling as potentially more relevant to capture the onset of focal seizures. Non-local effects were described by the lumped input parameter, *u*, rather than explicitly by inter-model connections. It is possible that long and short seizures could be differentiated earlier based on inter-channel connectivity patterns. Future work will focus on extending the estimation algorithm for non-locally connected neural regions. An inverse solution to the time-varying, multi-scale network problem is not trivial and is likely to require additional constraints. For example, structural MRI data may inform prior probabilities of connection strength [58]. Individual neural models can also be coupled within a larger scale network [15]. The approach taken by Schmidt et. al. (2016) can be adapted to set prior probabilities, or otherwise constrain the propagation of an assumed density (Kalman) filter.

The challenge of large-scale model inversion is relatively well understood [4, 26]. A more recent problem in EEG analysis is the challenge of dealing with very high dimensional data. This study involved separate dimensions for model parameters, seizures, patients, electrode channels, and time. Distilling insights from such a large dataset is computationally intensive. To provide some insight into this problem, the estimation results presented were 1.5TB in size. Generation of each figure can take up to a week to complete for all patients. The use of “big data” techniques for EEG are becoming more relevant to the study of epilepsy [62]. It is important that tools for large scale analysis of EEG are made clinically available. The model inversion technique presented in this work is generalizable and freely available (https://github.com/pkaroly/Data-Driven-Estimation).

It is important to note that the presented results are only valid insofar as the connectivity parameters of a neural model capture the relevant dynamics underlying seizure transitions. The use of neural mass model to investigate seizures has gained wide acceptance among epilepsy researchers [11, 63–65]. Tracking excitatory and inhibitory strengths within a network is considered highly relevant to understanding and treating seizures [66]. The ability to infer directional connections (differentiate between in-going and outgoing pyramidal connections) is also an important feature of model inversion compared with alternative graph inference measures. The estimation method was previously validated on simulated data [25]. Nevertheless, it is highly challenging to quantify the accuracy of the model reconstructions from real data, where there is no ground truth. The results showed that the difference between reconstructed and actual ECoG was small (Supplementary S1, S2 and S3 Figs). The consistency of results across many seizures provides evidence that the estimation can give overarching insight into mechanisms of patients’ seizures. It is our hope that this study provides a stepping stone towards a fully validated model inversion framework to guide the clinical management of epilepsy. Future experimental work should investigate whether modulating connectivity strengths in a stereotypical fashion does lead to different energy and/or duration of seizures, as predicted by the current analysis.

### Conclusion

This work provided a demonstration that the hidden local connectivity parameters of a neural mass model can be dynamically inferred from ECoG. Our results showed that seizures follow stereotypical pathways through parameter space. It is apparent that once a seizure has begun, a predefined sequence of states must be traversed before termination. For a subset of patients, there were two routes (short and long) to seizure termination. Short and long seizures began the same way but showed distinct offset mechanisms. Finally, the connectivity patterns at seizure onset showed common motifs across patients. These distinct sub-groups of onset mechanisms may suggest targeted treatment.

Techniques that unify neural mass models with data provide the means to address some of the unanswered hypotheses pertaining to epileptic dynamics. For example, theoretical studies have hypothesized that seizure trajectories are “innate”, or “repeatable” [13, 52]. The current results confirm that seizure pathways are indeed patient-specific and highly stereotyped. It has also been suggested that there are limited classes of onset mechanisms for seizures [43]. The current results show that there does appear to be a limited number of seizure onset “motifs” among patients. Finally, our group had previously hypothesized that long and short seizures reflect distinct cortical mechanisms [37]. The current results demonstrate that long and short seizures follow the same pathways but have different termination mechanisms. These results underscore the power of theoretical models to shed light on seizure mechanisms. It is our hope that these insights guide further modeling studies and may even prove to be directly translatable into clinical practice.

## Supporting information

S1 Appendix Neural model estimation. (PDF)

S1 Fig Mean squared error between measured and estimated ECoG. Distribution of average (across 16 channels) mean squared error of the estimated ECoG for all patients. Box plots show mean (circle), inter-quartile ranges (black square), 5%-95% ranges (black line), and outliers (black dots) over all seizures for that subject. Mean of the error distributions ranged from 0.2 to 0.9 mV (note that the mean amplitude of the measured ECoG signal ranges from approximately 25—100mV).
(PNG)

S2 Fig State estimation covariance. Distribution of average (across 16 channels) estimation error for the post-synaptic potential state variables. Covariance is expressed as a percentage of the estimate value. Each subplot shows the results for a given subject. Box plots show mean (circle), inter-quartile ranges (black square), 5%-95% ranges (black line), and outliers (black dots) over all seizures for that subject. Mean of the covariance distributions ranged from 2% to 16%.
(PNG)

S3 Fig Parameter estimation covariance. Distribution of average (across 16 channels) estimation error for the synaptic connectivity parameters. Covariance is expressed as a percentage of the estimate value. Each subplot shows the results for a given subject. Box plots show mean (circle), inter-quartile ranges (black square), 5%-95% ranges (black line), and outliers (black dots) over all seizures for that subject. Mean of the covariance distributions ranged from 0.1% to 10%.
(PNG)

S4 Fig Estimated changes in parameter trajectories during every seizure. Each subpanel represents the connectivity strength for all seizures (sorted by duration) from each patient. Parameters are expressed as a normalised percentage change from their pre-ictal background values (where 0 reflects no change from the pre-ictal period). Values are normalised, so -1 and 1 represent the minimal and maximal change for each individual parameter (i.e. absolute value comparisons between parameters cannot be made). The minimal and maximal parameter values were computed across all seizures for each individual patient. The pre-ictal period was defined from 2 minutes to 1 minute before seizure onset.
(PNG)

S5 Fig Average changes in parameter trajectories for long and short seizures. Each subpanel represents the mean connectivity strength (averaged across long and short seizures separately) from each patient (shading represents the 95% confidence bounds of the mean). Parameters are expressed as a percentage change from their pre-ictal background values (where 0 reflects no change from the pre-ictal period). The pre-ictal period was defined from 2 minutes to 1 minute before seizure onset. Significant (p < 0.05) differences between long and short trajectories are marked in black above each plot.
(PNG)

S6 Fig Significant changes in average parameter trajectories for all channels. Each subpanel represents the significance of changes in mean connectivity strength (averaged across seizures, x-axis) for all channels (y-axis) from each patient. Parameters were expressed as a percentage change from their pre-ictal background values (shown in Fig 5). Significant (p < 0.05) increase in connectivity strength is shown in red, and significant decrease is shown in blue. Columns from A-E show the connectivity parameters: A) external input to pyramidal neurons, B) inhibitory to pyramidal connectivity, C) excitatory to pyramidal connectivity, D) pyramidal to excitatory connectiviy, E) pyramidal to inhibitory connectiviy.
(PNG)

S7 Fig Correlation between connectivity and seizure duration. Correlation was measured between seizure duration and average connectivity strength taken 5s before seizure onset (Panel A), and 5s before seizure offset (Panel B). For each patient and parameter one coloured vertical bar is shown per channel (16 electrodes). Only significant correlation values are shown (p < 0.05). A Bonferonni correction for multiple comparisons was performed before computing significance, where the 5% significance level was divided by 60 (12 patients and 5 parameters).
(PNG)

S8 Fig Seizure energy for Patient 1. Signal energy during evolution of seizures, sorted by duration, from 10s before seizure onset (marked by arrowhead) to 10s after seizure termination (according to clinicians’ marking). Energy was computed for a 1s sliding window (50% overlap).
(TIF)

S9 Fig Seizure energy for Patient 2. Signal energy during evolution of seizures, sorted by duration, from 10s before seizure onset (marked by arrowhead) to 10s after seizure termination (according to clinicians’ marking). Energy was computed for a 1s sliding window (50% overlap).
(TIF)

S10 Fig Seizure energy for Patient 3. Signal energy during evolution of seizures, sorted by duration, from 10s before seizure onset (marked by arrowhead) to 10s after seizure termination (according to clinicians’ marking). Energy was computed for a 1s sliding window (50% overlap).
(TIF)

S11 Fig Seizure energy for Patient 4. Signal energy during evolution of seizures, sorted by duration, from 10s before seizure onset (marked by arrowhead) to 10s after seizure termination (according to clinicians’ marking). Energy was computed for a 1s sliding window (50% overlap).
(TIF)

S12 Fig Seizure energy for Patient 6. Signal energy during evolution of seizures, sorted by duration, from 10s before seizure onset (marked by arrowhead) to 10s after seizure termination (according to clinicians’ marking). Energy was computed for a 1s sliding window (50% overlap).
(TIF)

S13 Fig Seizure energy for Patient 7. Signal energy during evolution of seizures, sorted by duration, from 10s before seizure onset (marked by arrowhead) to 10s after seizure termination (according to clinicians’ marking). Energy was computed for a 1s sliding window (50% overlap).
(TIF)

S14 Fig Seizure energy for Patient 8. Signal energy during evolution of seizures, sorted by duration, from 10s before seizure onset (marked by arrowhead) to 10s after seizure termination (according to clinicians’ marking). Energy was computed for a 1s sliding window (50% overlap).
(TIF)

S15 Fig Seizure energy for Patient 9. Signal energy during evolution of seizures, sorted by duration, from 10s before seizure onset (marked by arrowhead) to 10s after seizure termination (according to clinicians’ marking). Energy was computed for a 1s sliding window (50% overlap).
(TIF)

S16 Fig Seizure energy for Patient 10. Signal energy during evolution of seizures, sorted by duration, from 10s before seizure onset (marked by arrowhead) to 10s after seizure termination (according to clinicians’ marking). Energy was computed for a 1s sliding window (50% overlap).
(TIF)

S17 Fig Seizure energy for Patient 11. Signal energy during evolution of seizures, sorted by duration, from 10s before seizure onset (marked by arrowhead) to 10s after seizure termination (according to clinicians’ marking). Energy was computed for a 1s sliding window (50% overlap).
(TIF)

S18 Fig Seizure energy for Patient 13. Signal energy during evolution of seizures, sorted by duration, from 10s before seizure onset (marked by arrowhead) to 10s after seizure termination (according to clinicians’ marking). Energy was computed for a 1s sliding window (50% overlap).
(TIF)

S19 Fig Seizure energy for Patient 15. Signal energy during evolution of seizures, sorted by duration, from 10s before seizure onset (marked by arrowhead) to 10s after seizure termination (according to clinicians’ marking). Energy was computed for a 1s sliding window (50% overlap).
(TIF)
