# Supplementary material for: Seizure pathways: A model-based investigation
Source: PLoS Comput Biol. 2018 Oct 11;14(10):e1006403. doi: 10.1371/journal.pcbi.1006403 (PMC6199000; doi:10.1371/journal.pcbi.1006403)
Supplement: S1 Appendix — (PDF) [file pcbi.1006403.s001.pdf]

S1 Appendix Neural Model Estimation

This section provides a description of the estimation method used to infer the states and parameters of the neural mass model from ECoG. The code used for this estimation, along with sample data and a tutorial can be found from `https://github.com/pkaroly/Data-Driven-Estimatio[illegible]` Note that this code includes a definition of all constant parameter values, as well as the steps taken to initalise the filter.

Representation of a neural region

The model of a cortical region can be written in the form

$$\dot{\mathbf{x}} = \mathbf{A}\mathbf{x} + \mathbf{B}\vec{\phi}(\mathbf{C}\mathbf{x}) \tag{1}$$

$$\mathbf{y} = \mathbf{H}\mathbf{x}, \tag{2}$$

where $\mathbf{x}$ is a vector of state variables, and $\mathbf{y}$ represents the measured ECoG.

We will now define the matrices, $\mathbf{A}$, $\mathbf{B}$, and $\mathbf{C}$. The matrix $\mathbf{A}$ encodes the dynamics induced by the membrane time constants. For N synapses, $\mathbf{A}$ has the block diagonal structure

$$\mathbf{A} = \mathrm{diag}\begin{pmatrix} \mathbf{\Psi}_1 & \dots & \mathbf{\Psi}_N \end{pmatrix},$$

where

$$\mathbf{\Psi}_n = \begin{bmatrix} 0 & 1 \\ -\frac{1}{\tau_{mn}} & -\frac{2}{\tau_{mn}} \end{bmatrix}.$$

The matrix of synaptic gains from internal inputs, $\mathbf{B}$, has the diagonal form

$$\mathbf{B} = \mathrm{diag}\begin{pmatrix} 0 & \alpha_1 & \dots & 0 & \alpha_{N_s} \end{pmatrix},$$

where, for instance, $\alpha_1 = \alpha_{xp}$ in our case.

The vector function $\phi(\cdot)$ has the following form

$$\vec{\phi}\left(\mathbf{C}\mathbf{x}\right) = \begin{bmatrix} 0 & g\left(\mathbf{c}_{2,:}\mathbf{x}\right) & \dots & 0 & g\left(\mathbf{c}_{N_x-2,:}\mathbf{x}\right) & 0 & u \end{bmatrix}^\top, \tag{3}$$

where $g()$ is the sigmoid function (defined in the main text), and u is a constant, non-local input to the pyramidal population. The adjacency matrix, $\mathbf{C}$, defines the connectivity structure of the model. It is a matrix of zeros or ones that specifies all the connections between the cell population types (excluding external inputs) that has the block structure

$$\mathbf{C} = \begin{bmatrix} 0 & 0 & \dots & 0 & 0 \\ \mathbf{c}_{2,1} & 0 & & \mathbf{c}_{2,N_x-1} & 0 \\ \vdots & & \ddots & & \vdots \\ 0 & 0 & & 0 & 0 \\ \mathbf{c}_{N_x,1} & 0 & & \mathbf{c}_{N_x,N_x-1} & 0 \end{bmatrix}.$$

Estimation filter for the augmented neural model

Our augmented state-space model is

$$\boldsymbol{\xi}_t = \mathbf{A}_\theta \boldsymbol{\xi}_{t-1} + \mathbf{B}_\theta \phi(\mathbf{C}_\theta \boldsymbol{\xi}_{t-1}) + \mathbf{w}_{t-1}. \tag{4}$$

The aim of the filter is to estimate the most likely sequences of states, $\hat{\boldsymbol{\xi}}_t^+$, and the associated error covariances, $\hat{\mathbf{P}}_t^+$, given by

$$\hat{\boldsymbol{\xi}}_t^+ = \mathbb{E}\left[\boldsymbol{\xi}_t | \mathbf{y}_1, \mathbf{y}_2, \ldots, \mathbf{y}_t\right] \tag{5}$$

$$\hat{\mathbf{P}}_t^+ = \mathbb{E}\left[\left(\boldsymbol{\xi}_t - \hat{\boldsymbol{\xi}}_t^+\right)\left(\boldsymbol{\xi}_t - \hat{\boldsymbol{\xi}}_t^+\right)^\top\right], \tag{6}$$

which are known as the a posteriori state estimate and state estimate covariance, respectively. These values are obtained by corecting the a priori state estiamate, $\hat{\boldsymbol{\xi}}_t^-$ and covariance, $\hat{\mathbf{P}}_t^-$. In this work we applied a semi-analytic solution to compute the a priori predictions. The a priori state estimate is given by

$$\begin{aligned}\hat{\boldsymbol{\xi}}_t^- &= \mathbb{E}\left[\boldsymbol{\xi}_t | \mathbf{y}_1, \mathbf{y}_2, \ldots, \mathbf{y}_{t-1}\right] \\ &= \mathbb{E}\left[\mathbf{A}\boldsymbol{\xi}_{t-1} + \mathbf{B}\phi(\boldsymbol{\xi}_{t-1}) + \mathbf{w}_t\right] \\ &= \mathbf{A}\hat{\boldsymbol{\xi}}_{t-1}^+ + \mathbf{B}\hat{\boldsymbol{\phi}}_{t-1},\end{aligned} \tag{7}$$

where the vectors (note the square root is element-wise and $\circ$ is the Hadamard product)

$$\begin{aligned}\hat{\boldsymbol{\phi}}_{t-1} &= \frac{1}{2}\left(\operatorname{erf}\left(\boldsymbol{\beta} \circ \boldsymbol{\sigma}^{-1/2}\right) + 1\right) \\ \boldsymbol{\beta}_t &= \mathbf{C}`\hat{\boldsymbol{\xi}}_{t-1}^+ - v_0 \\ \boldsymbol{\sigma}_t &= 2\left(\operatorname{diag}\left(\mathbf{C}\mathbf{P}_{t-1}^+\mathbf{C}^\top\right) + \varsigma^2\right).\end{aligned} \tag{8}$$

The a priori state estimate error covariance is

$$\begin{aligned}
\hat{\mathbf{P}}_t^- =& \mathbb{E}\left[\left(\boldsymbol{\xi}_t - \hat{\boldsymbol{\xi}}_t^-\right)\left(\boldsymbol{\xi}_t - \hat{\boldsymbol{\xi}}_t^-\right)^\top\right] \\
=& \mathbb{E}\left[\left(\mathbf{A}\boldsymbol{\xi}_{t-1} + \mathbf{B}\phi(\mathbf{C}\boldsymbol{\xi}_{t-1}) + \mathbf{w}_{t-1} - \left(\mathbf{A}\hat{\boldsymbol{\xi}}_{t-1}^+ + \mathbf{B}\hat{\boldsymbol{\phi}}_{t-1}\right)\right)(\,\cdot\,)^\top\right] \\
=& \mathbf{A}\hat{\mathbf{P}}_{t-1}^+\mathbf{A}^\top + \mathbf{B}\mathbb{E}\left[\phi(\boldsymbol{\xi}_{t-1})\phi^\top(\boldsymbol{\xi}_{t-1})\right]\mathbf{B}^\top + \mathbf{Q} - \mathbf{B}\hat{\boldsymbol{\phi}}_{t-1}\hat{\boldsymbol{\phi}}_{t-1}^\top\mathbf{B}^\top + \boldsymbol{\Phi}_{t-1} + \boldsymbol{\Phi}_{t-1}^\top, \qquad (9)
\end{aligned}$$

where

$$\begin{aligned}
\boldsymbol{\Phi}_{t-1} =& \mathbf{A}\mathbb{E}\left[\boldsymbol{\xi}_{t-1}\phi^\top(\mathbf{C}\boldsymbol{\xi}_{t-1})\right]\mathbf{B}^\top - \mathbf{A}\hat{\boldsymbol{\xi}}_{t-1}^+\mathbb{E}\left[\boldsymbol{\phi}^\top\left(\mathbf{C}\boldsymbol{\xi}_{t-1}\right)\right]\mathbf{B}^\top \\
=& \mathbf{A}\left(\mathbf{P}_{t-1}^+\mathbf{C}^\top \circ \mathbf{1} \times \boldsymbol{\Lambda}^\top\right)\mathbf{B}^\top \qquad (10)
\end{aligned}$$

$$\boldsymbol{\Lambda} = (\pi\boldsymbol{\sigma})^{-1/2}\exp\left(-\boldsymbol{\beta}\circ\boldsymbol{\beta}\circ\boldsymbol{\sigma}^{-1}\right). \qquad (11)$$

We can analytically calculate all the elements of $\hat{\mathbf{P}}_t^-$ except for $\mathbb{E}\left[\phi(\boldsymbol{\xi}_{t-1})\phi^\top(\boldsymbol{\xi}_{t-1})\right]$. Instead, we use a precise solution from the multivariate Gaussian cumulative density function [1]. The elements, indexed by i and j, of the matrix resulting from evaluating the expectation are equivalent to the probabilities of the bivariate Gaussians

$$\mathbb{E}\left[\boldsymbol{\phi}\left(\mathbf{C}\boldsymbol{\xi}_{t-1}\right)\boldsymbol{\phi}^\top\left(\mathbf{C}\boldsymbol{\xi}_{t-1}\right)\right]_{ij} = P\left(x>0, y>0\right), \qquad (12)$$

where $(x,y)^\top \sim \mathcal{N}\left(\boldsymbol{\mu}, \boldsymbol{\Sigma}\right)$ and

$$\begin{aligned}
\boldsymbol{\mu} =& -\left[\left(\mathbf{C}\hat{\boldsymbol{\xi}}_{t-1}^+\right)_i, \left(\mathbf{C}\hat{\boldsymbol{\xi}}_{t-1}^+\right)_j\right]^\top \\
\boldsymbol{\Sigma} =& \begin{bmatrix} \left(\operatorname{diag}\left(\mathbf{C}\hat{\mathbf{P}}_{t-1}^+\mathbf{C}^\top\right) + \varsigma^2\right)_i & \left(\mathbf{C}\hat{\mathbf{P}}_{t-1}^+\mathbf{C}^\top\right)_{ij} \\ \left(\mathbf{C}\hat{\mathbf{P}}_{t-1}^+\mathbf{C}^\top\right)_{ij} & \left(\operatorname{diag}\left(\mathbf{C}\hat{\mathbf{P}}_{t-1}^+\mathbf{C}^\top\right) + \varsigma^2\right)_j \end{bmatrix}.
\end{aligned}$$

These probabilities can be computed easily in Matlab using, where each element is `mvncdf` $(\mathbf{0}, \boldsymbol{\mu}, \boldsymbol{\Sigma})$.

Filter initialization

We used the average and covariance of simulated data to intialise $\hat{\boldsymbol{\xi}}_0^+$ and $\hat{\mathbf{P}}_0^+$ (the *a priori* mean and covariance for time $t = 0$). For the model uncertainty we used small constant values ($\approx 5\mu$V), which prevents the filter converging, and enables new measurements to continue to influence the estimation. For the measurement noise we used a value of 1mV. More technical details of filter initialisation can be found online (`https://github.com/pkaroly/Data-Driven-Estimation`).

References

1. Genz, A. Numerical computation of rectangular bivariate and trivariate normal and t probabilities. *Statistics and Computing* **14**, 251–260 (2004).
