# Supplementary figures and images for: Seizure pathways: A model-based investigation
Source: PLoS Comput Biol. 2018 Oct 11;14(10):e1006403. doi: 10.1371/journal.pcbi.1006403 (PMC6199000; doi:10.1371/journal.pcbi.1006403)

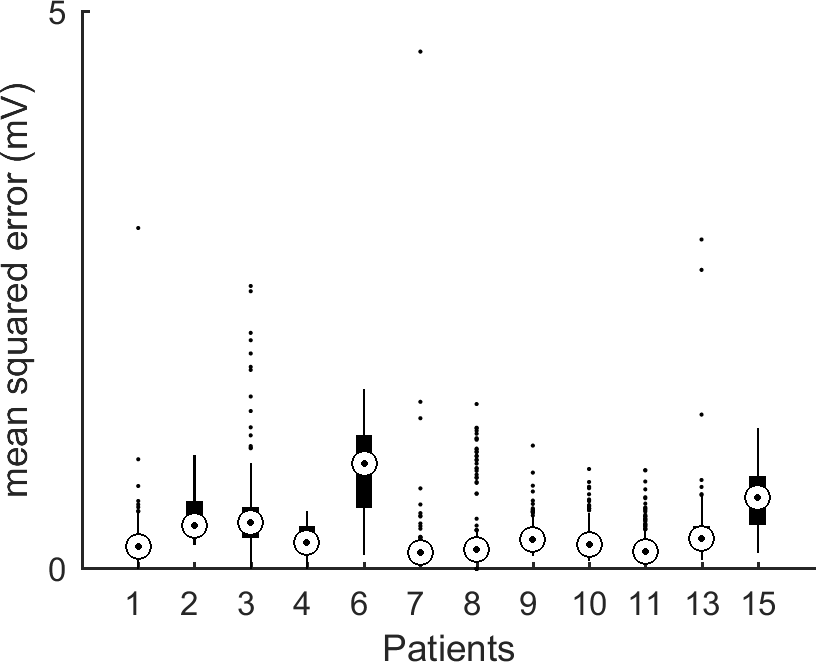

Supplement: S1 Fig — Distribution of average (across 16 channels) mean squared error of the estimated ECoG for all patients. Box plots show mean (circle), inter-quartile ranges (black square), 5%-95% ranges (black line), and outliers (black dots) over all seizures for that subject. Mean of the error distributions ranged from 0.2 to 0.9 mV (note that the mean amplitude of the measured ECoG signal ranges from approximately 25—100mV). (PNG) [file pcbi.1006403.s002.png]

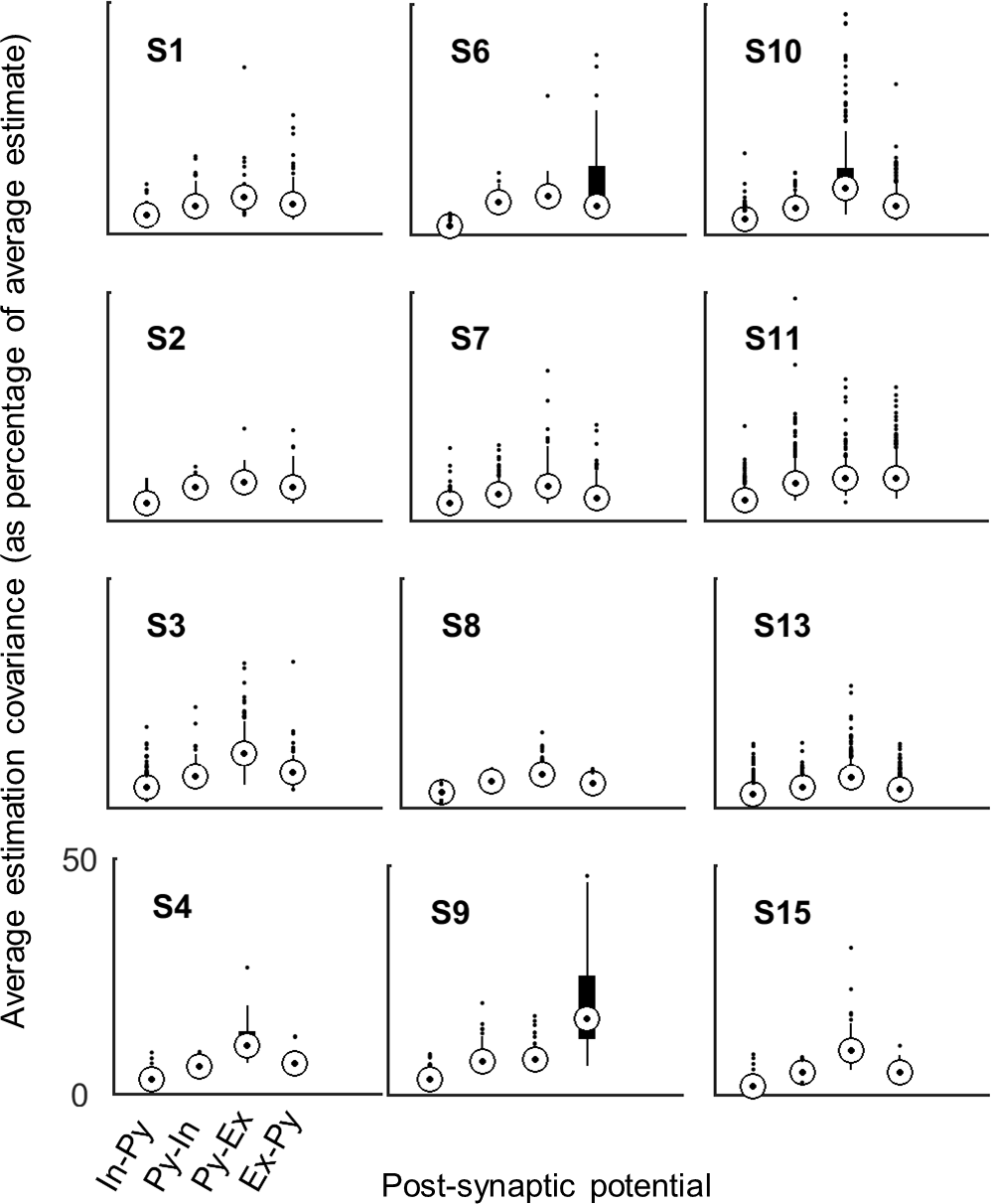

Supplement: S2 Fig — Distribution of average (across 16 channels) estimation error for the post-synaptic potential state variables. Covariance is expressed as a percentage of the estimate value. Each subplot shows the results for a given subject. Box plots show mean (circle), inter-quartile ranges (black square), 5%-95% ranges (black line), and outliers (black dots) over all seizures for that subject. Mean of the covariance distributions ranged from 2% to 16%. (PNG) [file pcbi.1006403.s003.png]

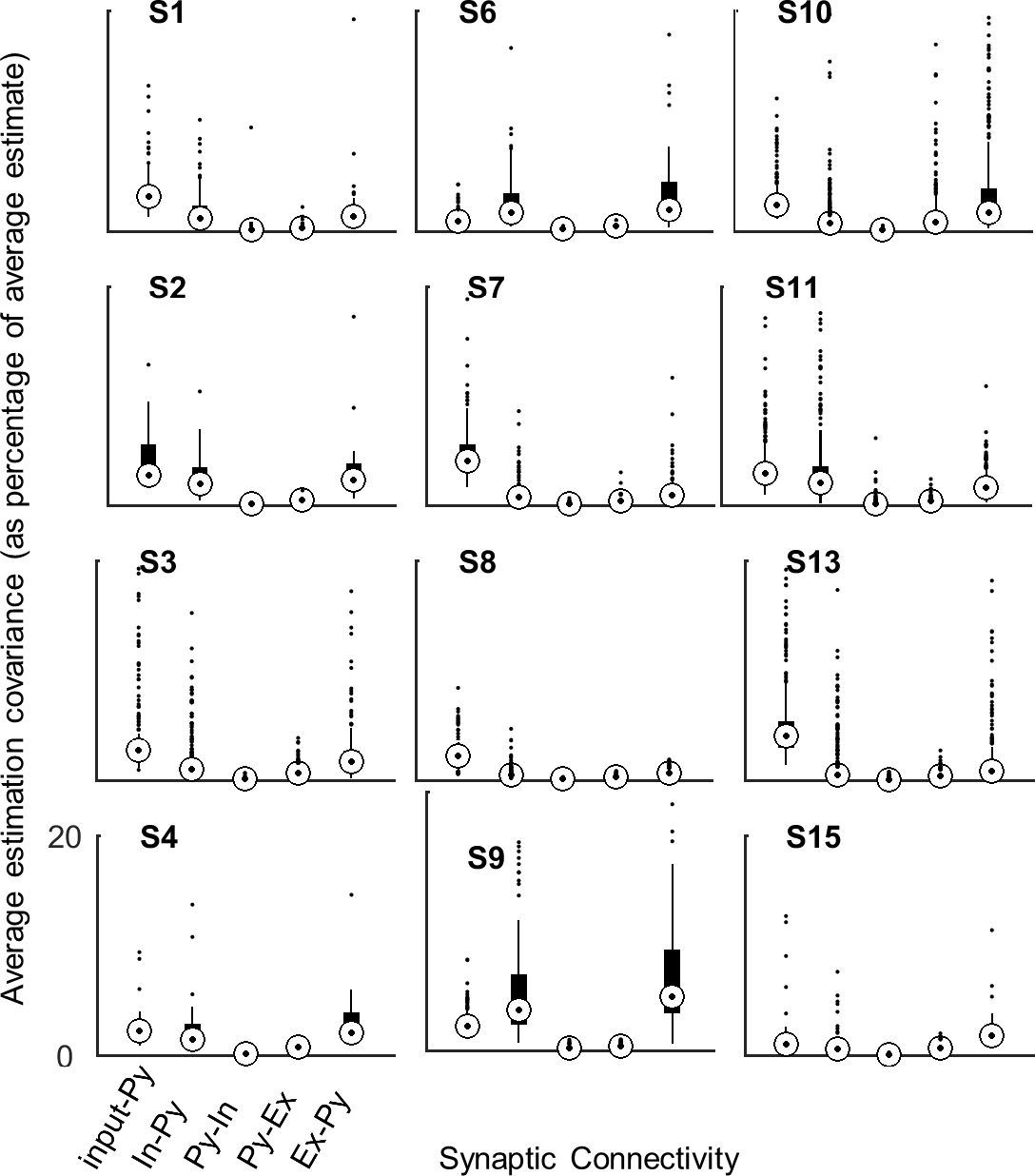

Supplement: S3 Fig — Distribution of average (across 16 channels) estimation error for the synaptic connectivity parameters. Covariance is expressed as a percentage of the estimate value. Each subplot shows the results for a given subject. Box plots show mean (circle), inter-quartile ranges (black square), 5%-95% ranges (black line), and outliers (black dots) over all seizures for that subject. Mean of the covariance distributions ranged from 0.1% to 10%. (PNG) [file pcbi.1006403.s004.png]

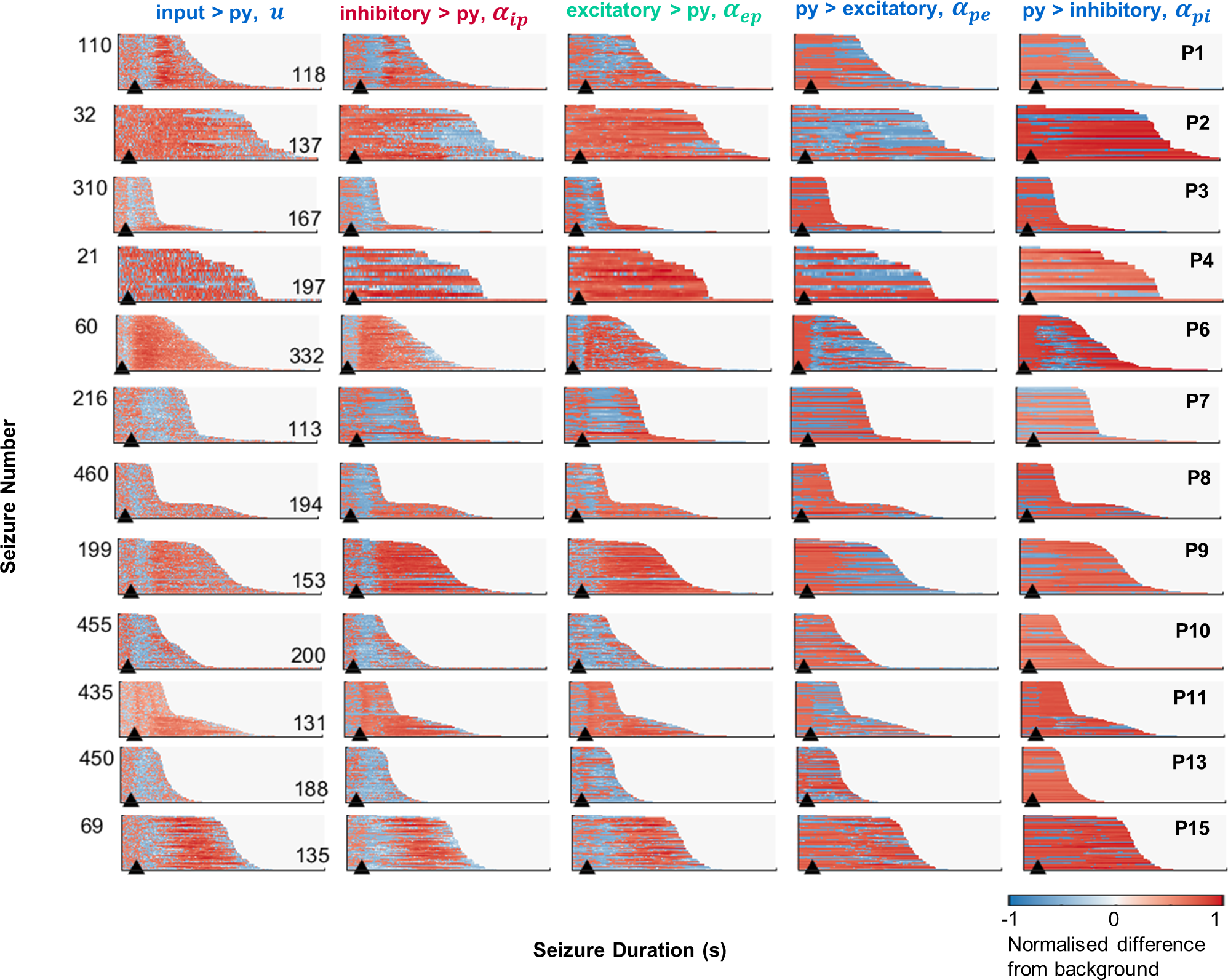

Supplement: S4 Fig — Each subpanel represents the connectivity strength for all seizures (sorted by duration) from each patient. Parameters are expressed as a normalised percentage change from their pre-ictal background values (where 0 reflects no change from the pre-ictal period). Values are normalised, so -1 and 1 represent the minimal and maximal change for each individual parameter (i.e. absolute value comparisons between parameters cannot be made). The minimal and maximal parameter values were computed across all seizures for each individual patient. The pre-ictal period was defined from 2 minutes to 1 minute before seizure onset. (PNG) [file pcbi.1006403.s005.png]

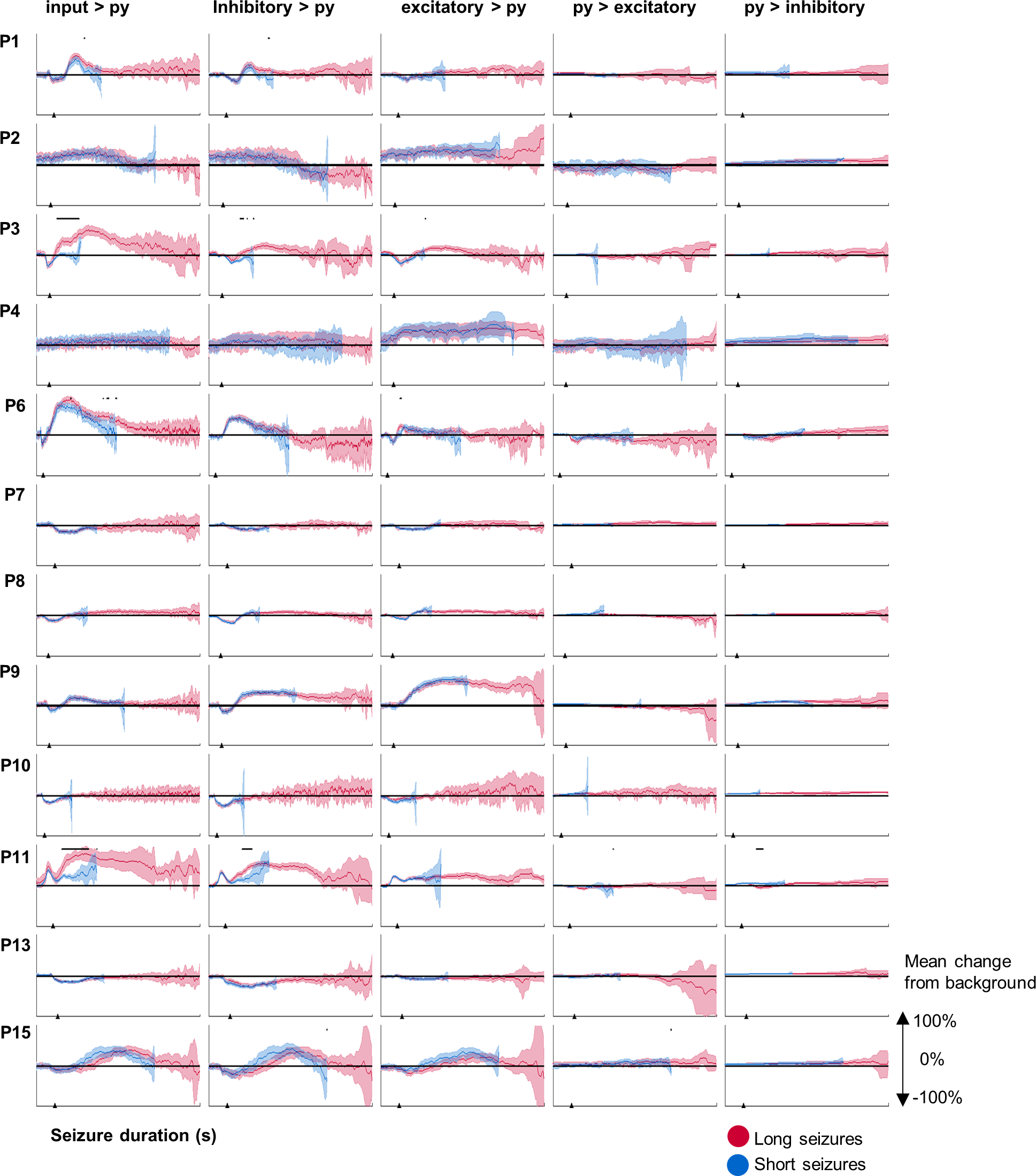

Supplement: S5 Fig — Each subpanel represents the mean connectivity strength (averaged across long and short seizures separately) from each patient (shading represents the 95% confidence bounds of the mean). Parameters are expressed as a percentage change from their pre-ictal background values (where 0 reflects no change from the pre-ictal period). The pre-ictal period was defined from 2 minutes to 1 minute before seizure onset. Significant (p < 0.05) differences between long and short trajectories are marked in black above each plot. (PNG) [file pcbi.1006403.s006.png]

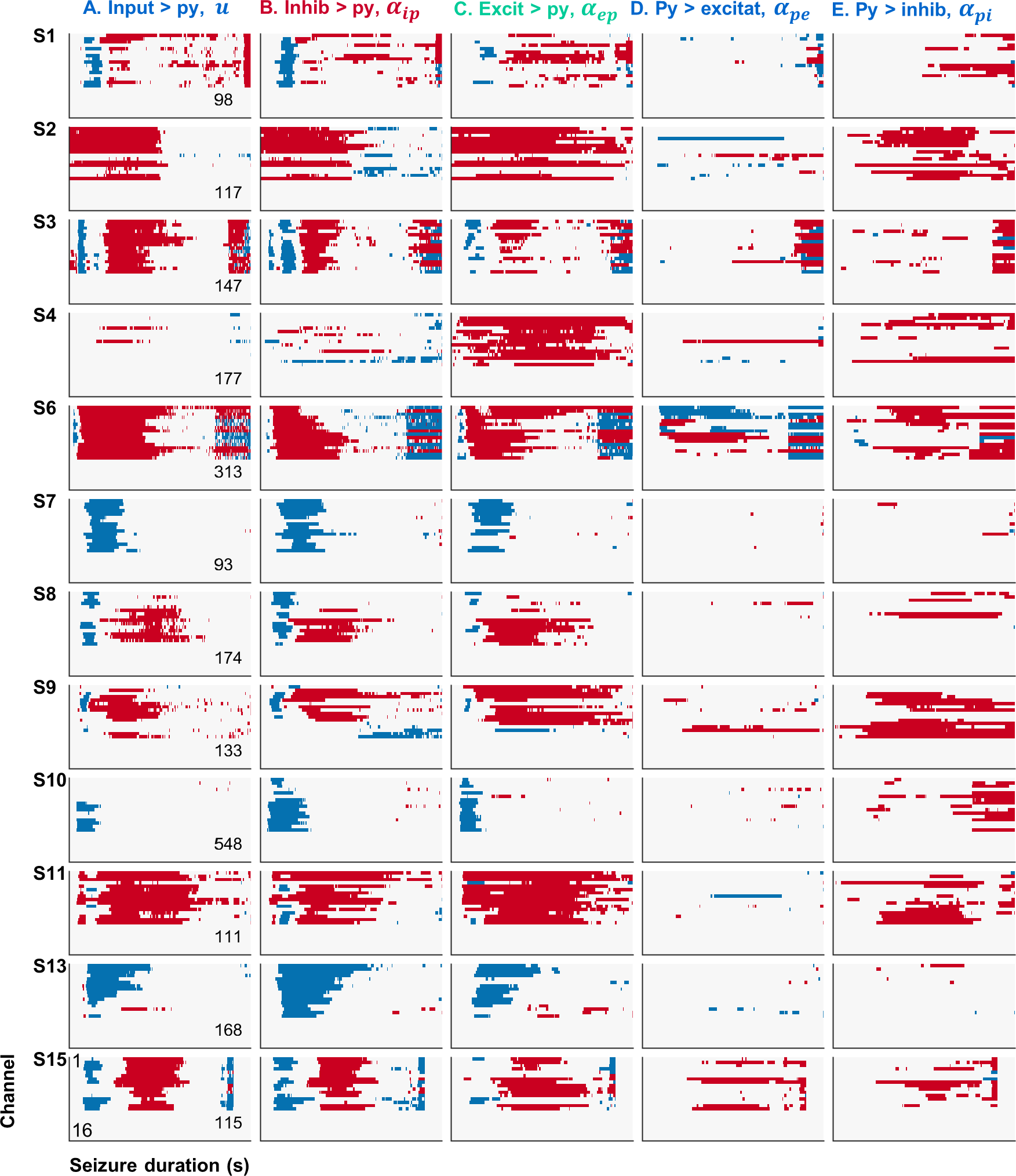

Supplement: S6 Fig — Each subpanel represents the significance of changes in mean connectivity strength (averaged across seizures, x-axis) for all channels (y-axis) from each patient. Parameters were expressed as a percentage change from their pre-ictal background values (shown in Fig 5). Significant (p < 0.05) increase in connectivity strength is shown in red, and significant decrease is shown in blue. Columns from A-E show the connectivity parameters: A) external input to pyramidal neurons, B) inhibitory to pyramidal connectivity, C) excitatory to pyramidal connectivity, D) pyramidal to excitatory connectiviy, E) pyramidal to inhibitory connectiviy. (PNG) [file pcbi.1006403.s007.png]

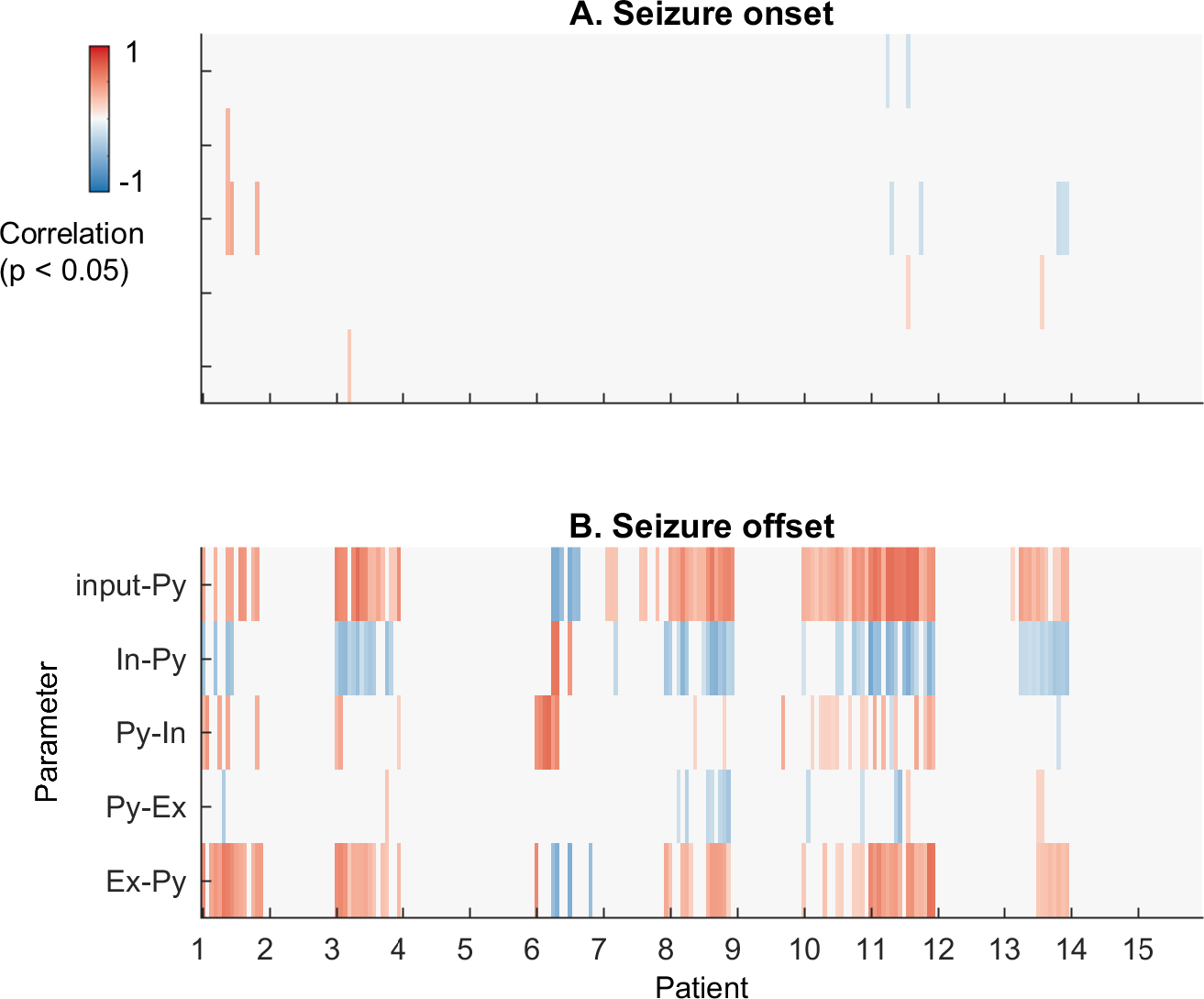

Supplement: S7 Fig — Correlation was measured between seizure duration and average connectivity strength taken 5s before seizure onset (Panel A), and 5s before seizure offset (Panel B). For each patient and parameter one coloured vertical bar is shown per channel (16 electrodes). Only significant correlation values are shown (p < 0.05). A Bonferonni correction for multiple comparisons was performed before computing significance, where the 5% significance level was divided by 60 (12 patients and 5 parameters). (PNG) [file pcbi.1006403.s008.png]

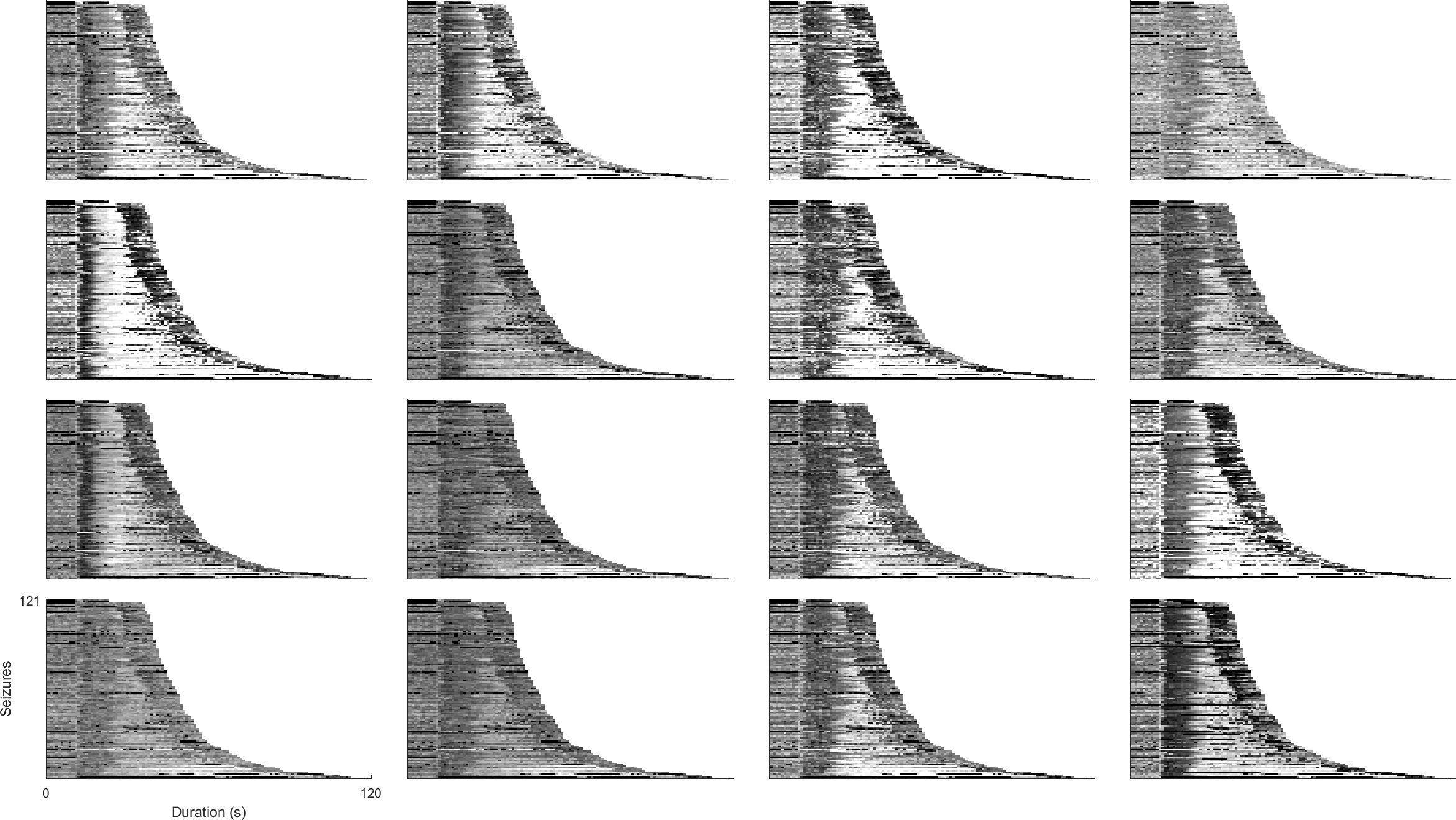

Supplement: S8 Fig — Signal energy during evolution of seizures, sorted by duration, from 10s before seizure onset (marked by arrowhead) to 10s after seizure termination (according to clinicians’ marking). Energy was computed for a 1s sliding window (50% overlap). (TIF) [file pcbi.1006403.s009.tif]

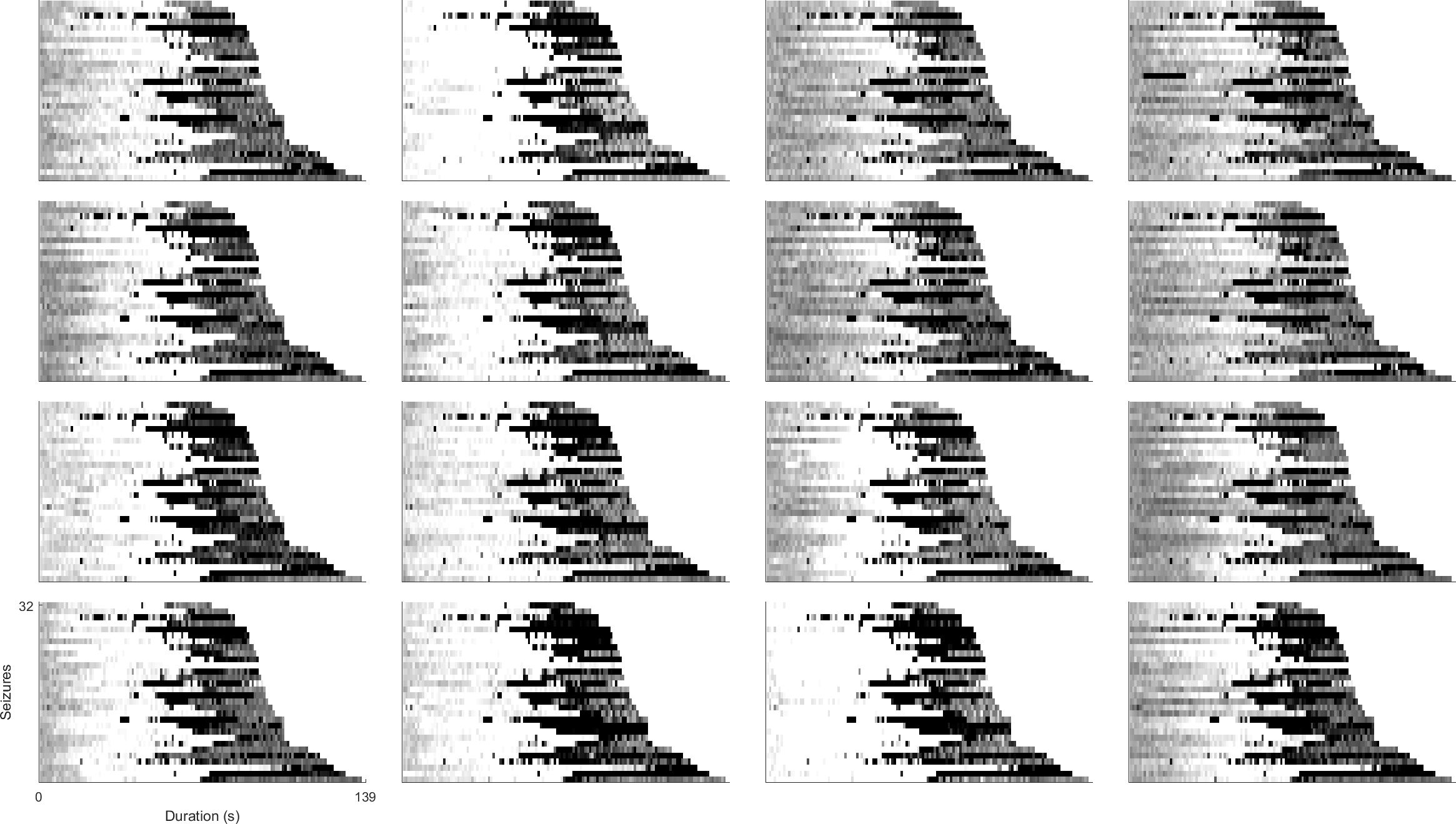

Supplement: S9 Fig — Signal energy during evolution of seizures, sorted by duration, from 10s before seizure onset (marked by arrowhead) to 10s after seizure termination (according to clinicians’ marking). Energy was computed for a 1s sliding window (50% overlap). (TIF) [file pcbi.1006403.s010.tif]

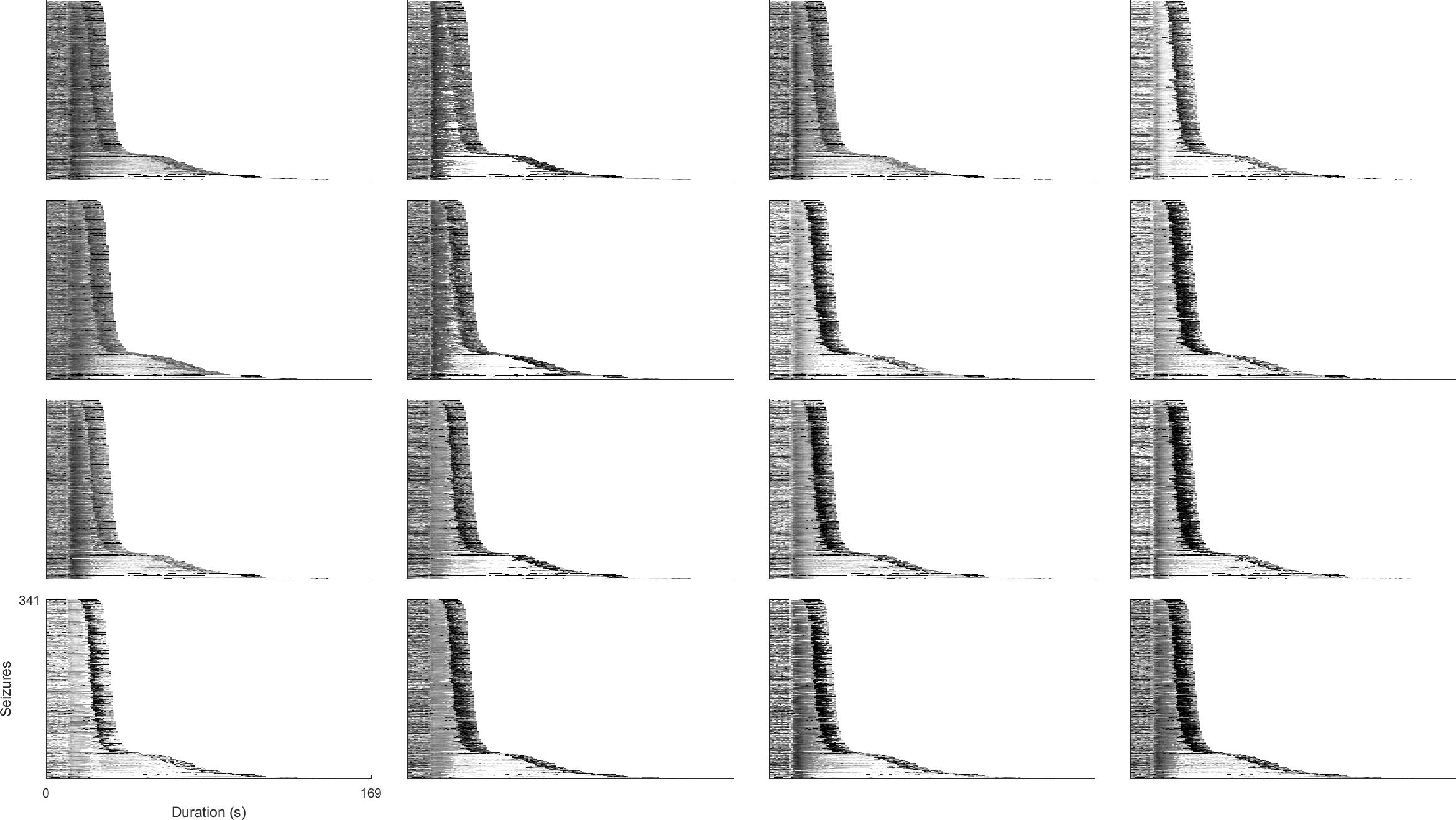

Supplement: S10 Fig — Signal energy during evolution of seizures, sorted by duration, from 10s before seizure onset (marked by arrowhead) to 10s after seizure termination (according to clinicians’ marking). Energy was computed for a 1s sliding window (50% overlap). (TIF) [file pcbi.1006403.s011.tif]

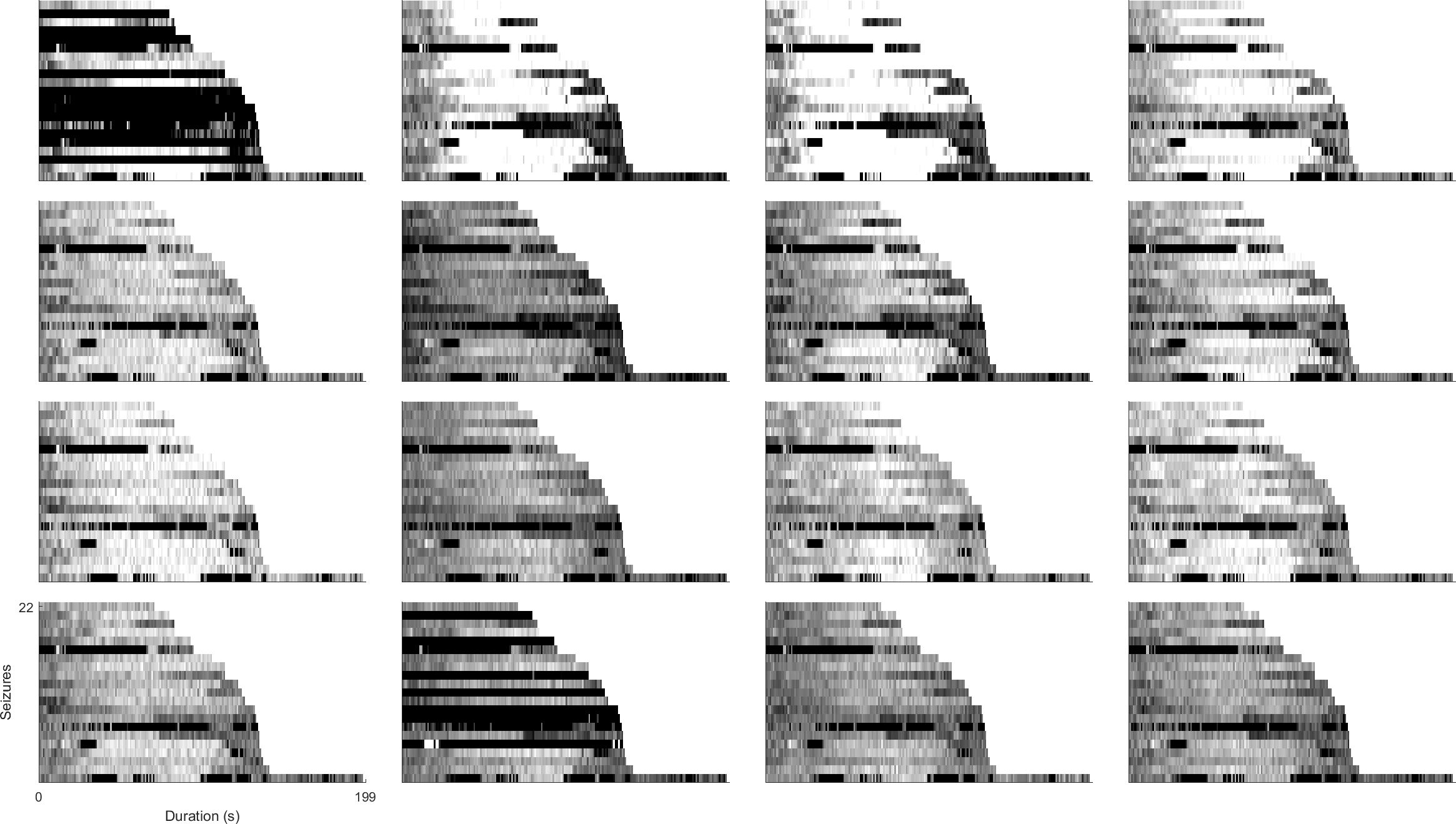

Supplement: S11 Fig — Signal energy during evolution of seizures, sorted by duration, from 10s before seizure onset (marked by arrowhead) to 10s after seizure termination (according to clinicians’ marking). Energy was computed for a 1s sliding window (50% overlap). (TIF) [file pcbi.1006403.s012.tif]

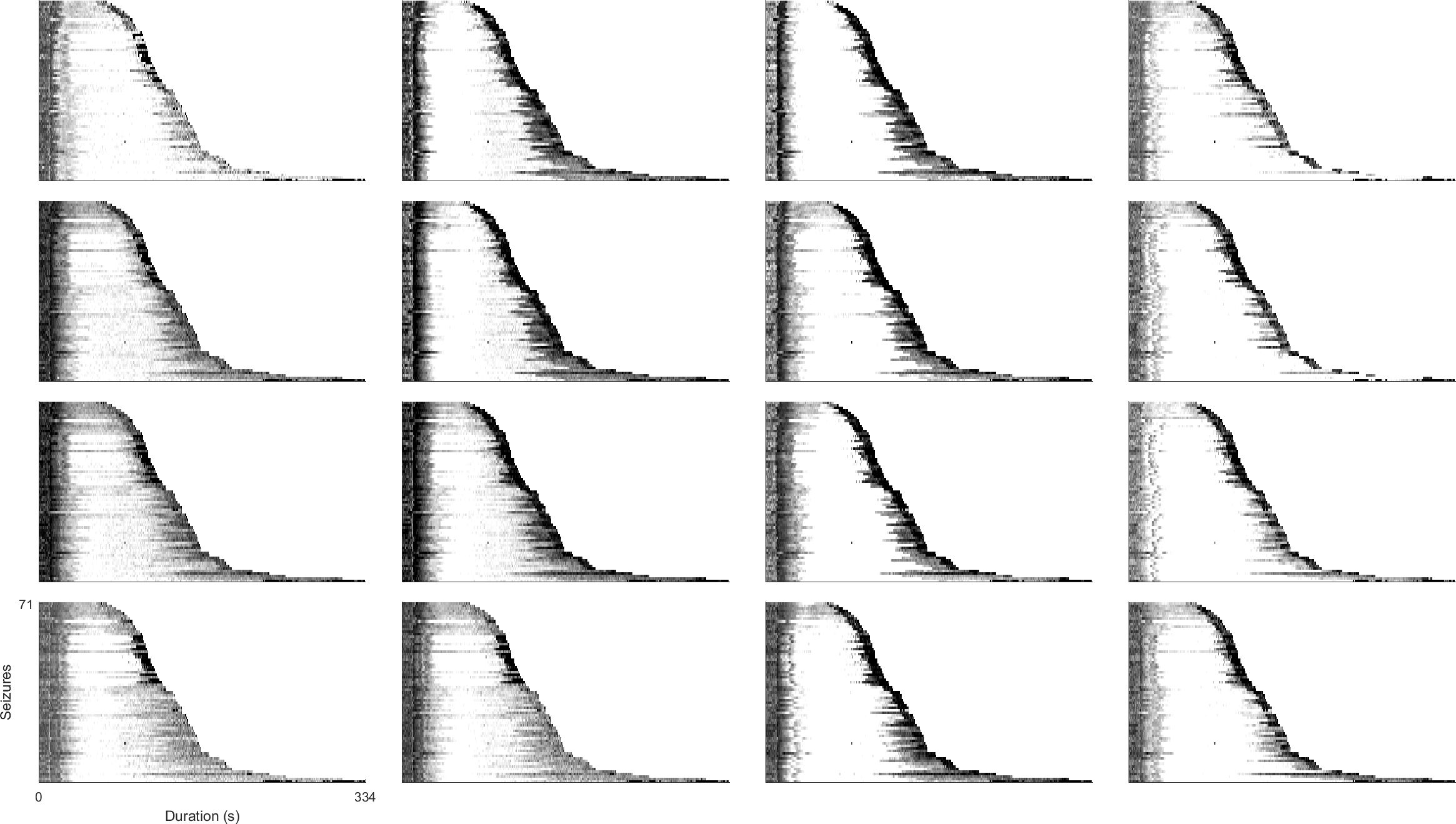

Supplement: S12 Fig — Signal energy during evolution of seizures, sorted by duration, from 10s before seizure onset (marked by arrowhead) to 10s after seizure termination (according to clinicians’ marking). Energy was computed for a 1s sliding window (50% overlap). (TIF) [file pcbi.1006403.s013.tif]

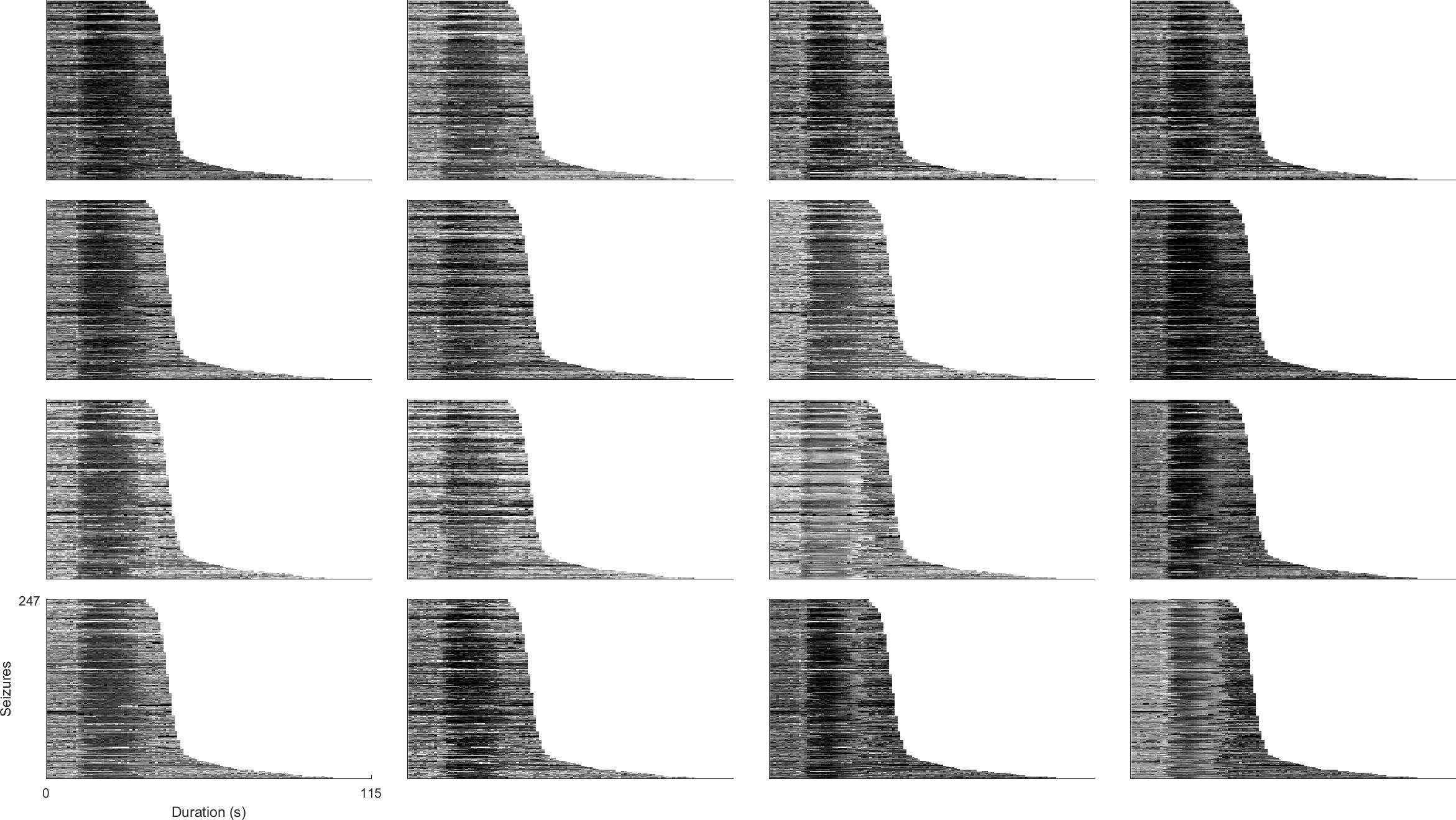

Supplement: S13 Fig — Signal energy during evolution of seizures, sorted by duration, from 10s before seizure onset (marked by arrowhead) to 10s after seizure termination (according to clinicians’ marking). Energy was computed for a 1s sliding window (50% overlap). (TIF) [file pcbi.1006403.s014.tif]

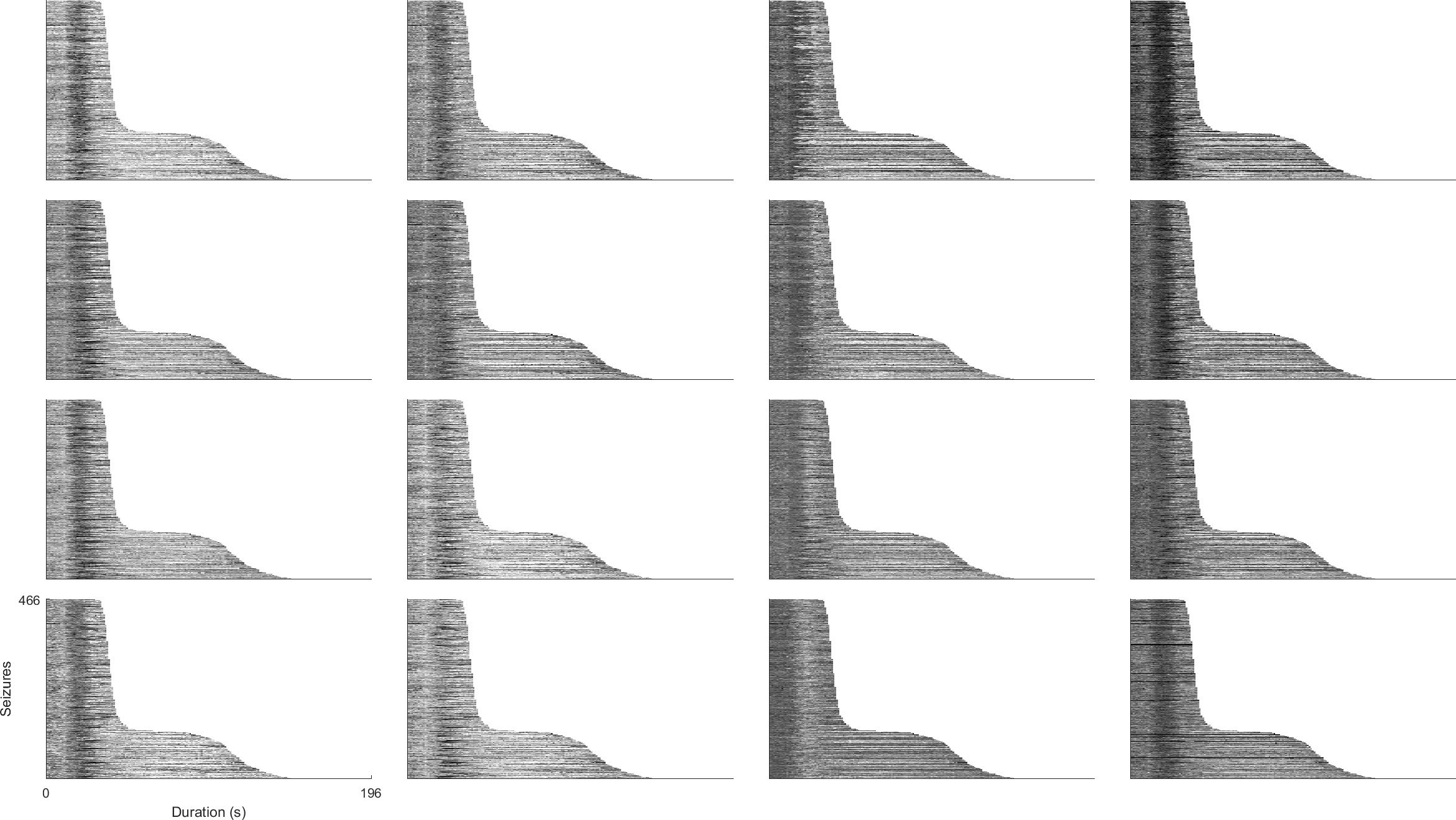

Supplement: S14 Fig — Signal energy during evolution of seizures, sorted by duration, from 10s before seizure onset (marked by arrowhead) to 10s after seizure termination (according to clinicians’ marking). Energy was computed for a 1s sliding window (50% overlap). (TIF) [file pcbi.1006403.s015.tif]

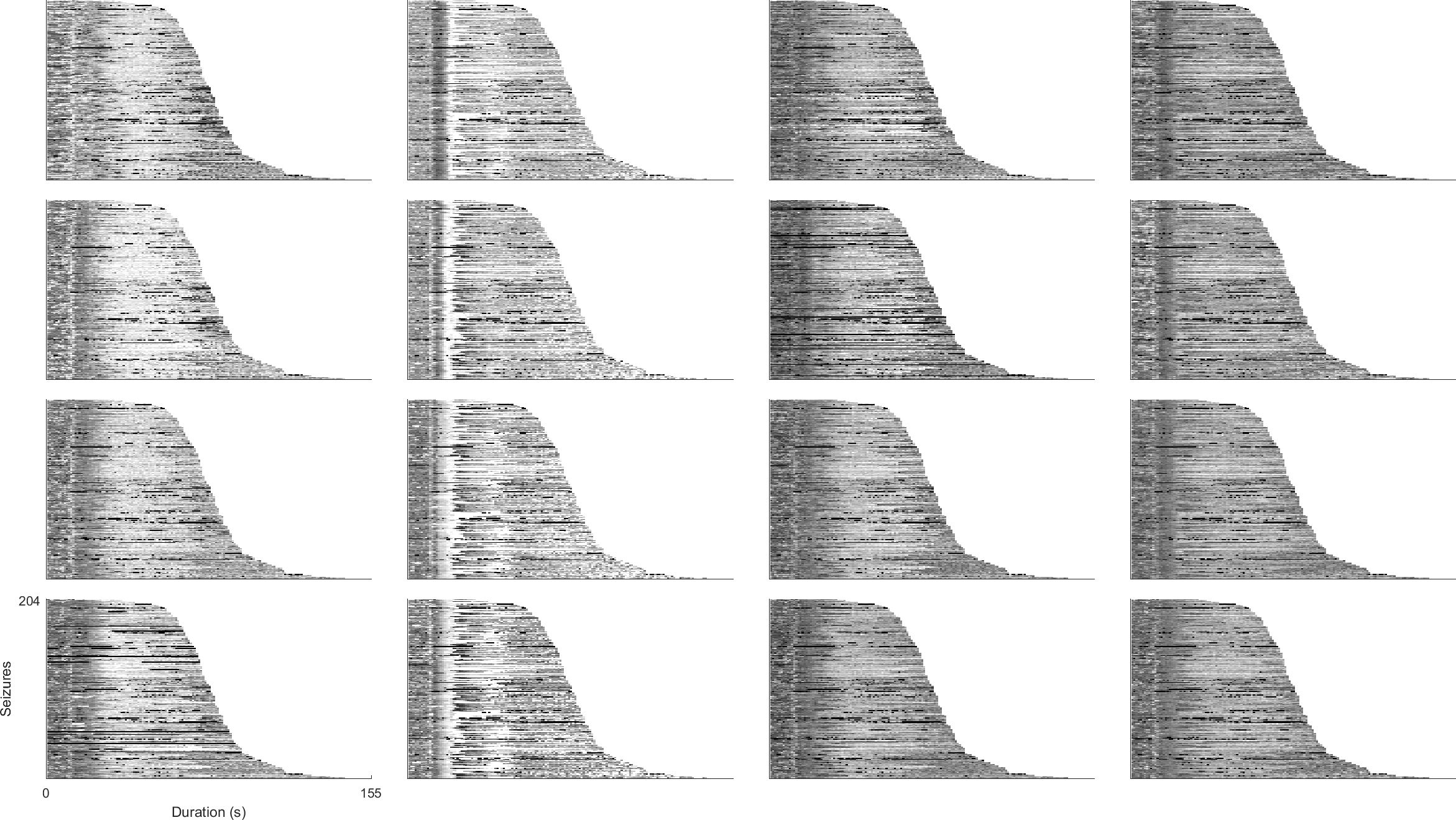

Supplement: S15 Fig — Signal energy during evolution of seizures, sorted by duration, from 10s before seizure onset (marked by arrowhead) to 10s after seizure termination (according to clinicians’ marking). Energy was computed for a 1s sliding window (50% overlap). (TIF) [file pcbi.1006403.s016.tif]

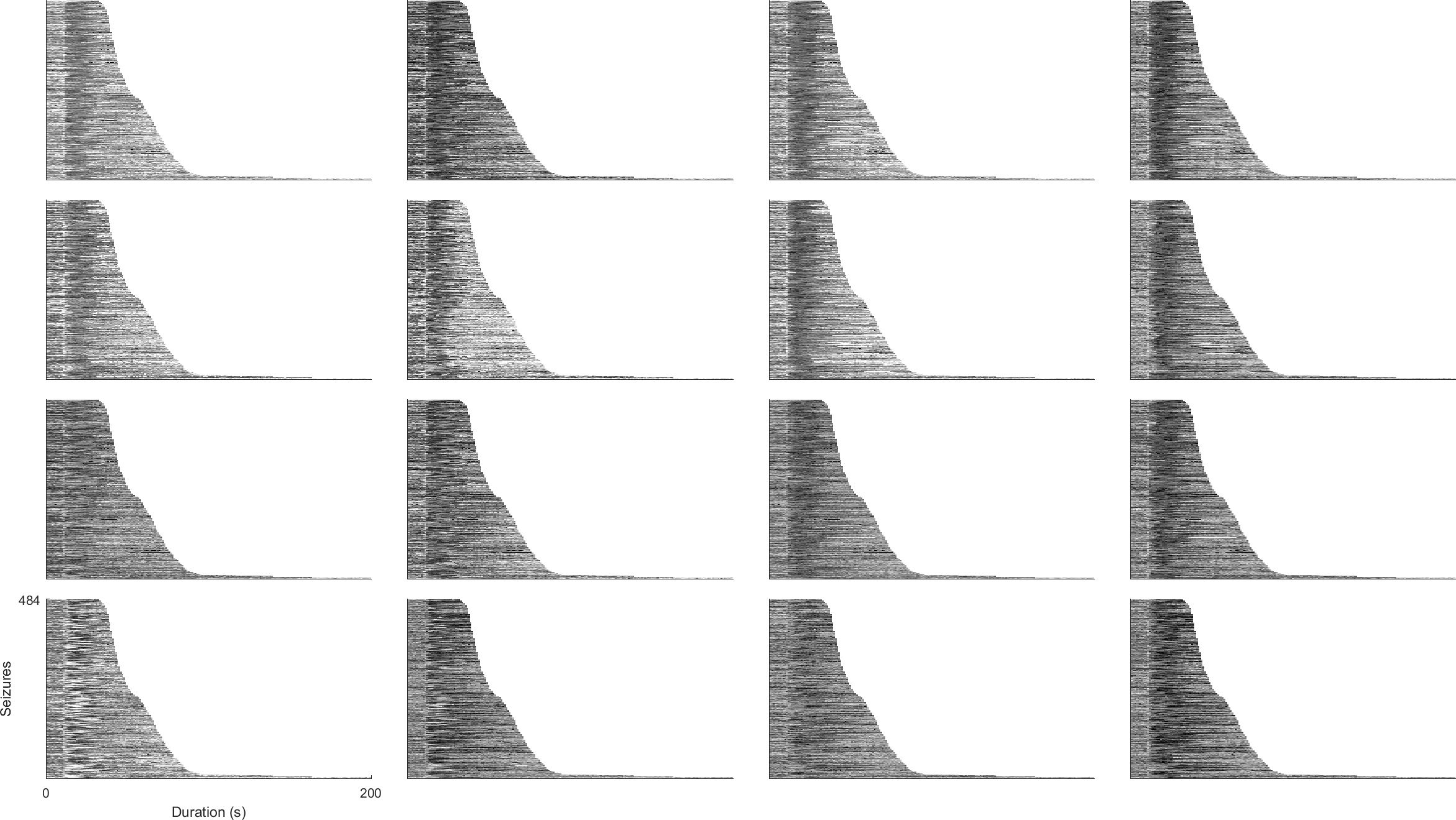

Supplement: S16 Fig — Signal energy during evolution of seizures, sorted by duration, from 10s before seizure onset (marked by arrowhead) to 10s after seizure termination (according to clinicians’ marking). Energy was computed for a 1s sliding window (50% overlap). (TIF) [file pcbi.1006403.s017.tif]

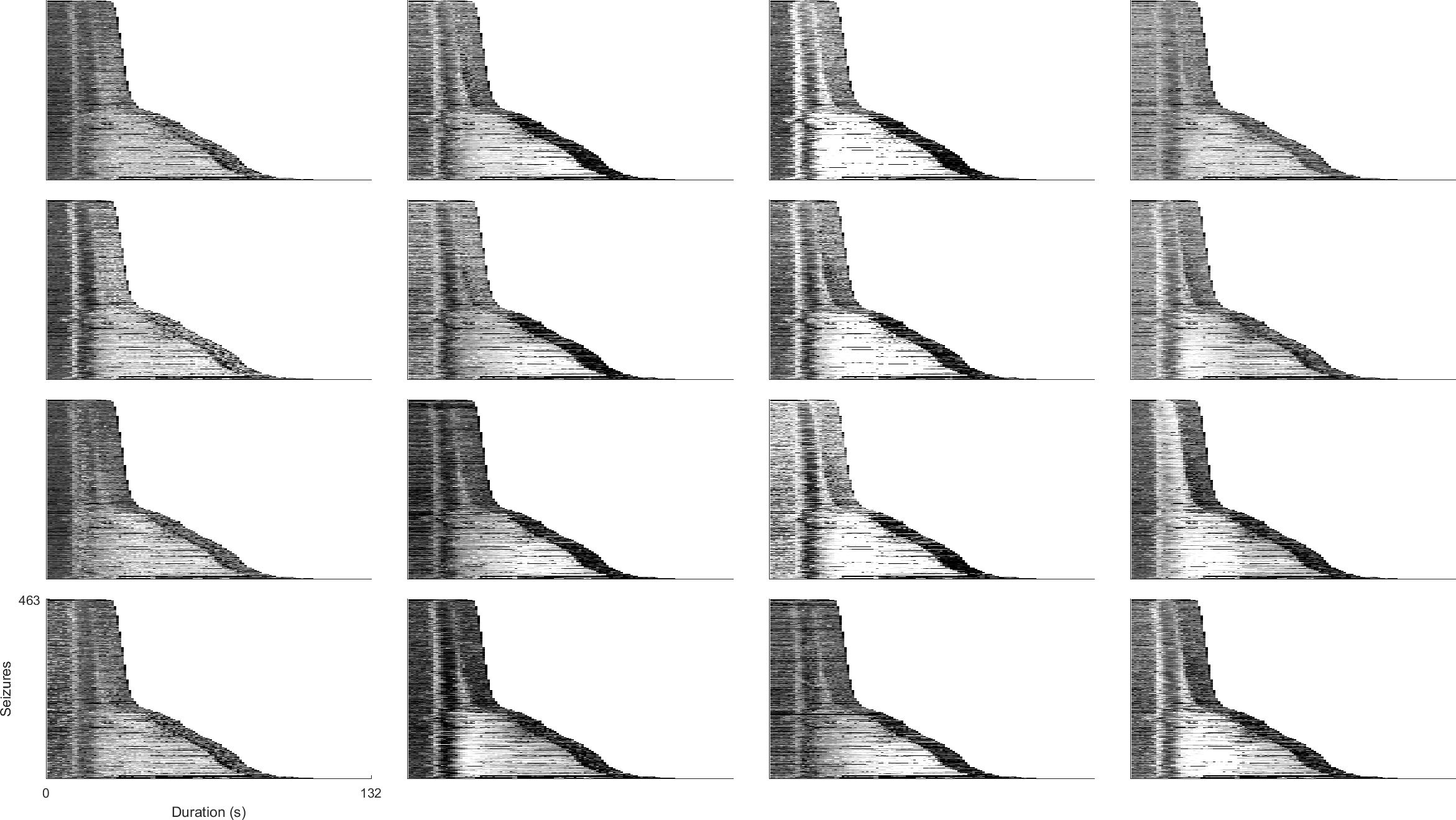

Supplement: S17 Fig — Signal energy during evolution of seizures, sorted by duration, from 10s before seizure onset (marked by arrowhead) to 10s after seizure termination (according to clinicians’ marking). Energy was computed for a 1s sliding window (50% overlap). (TIF) [file pcbi.1006403.s018.tif]

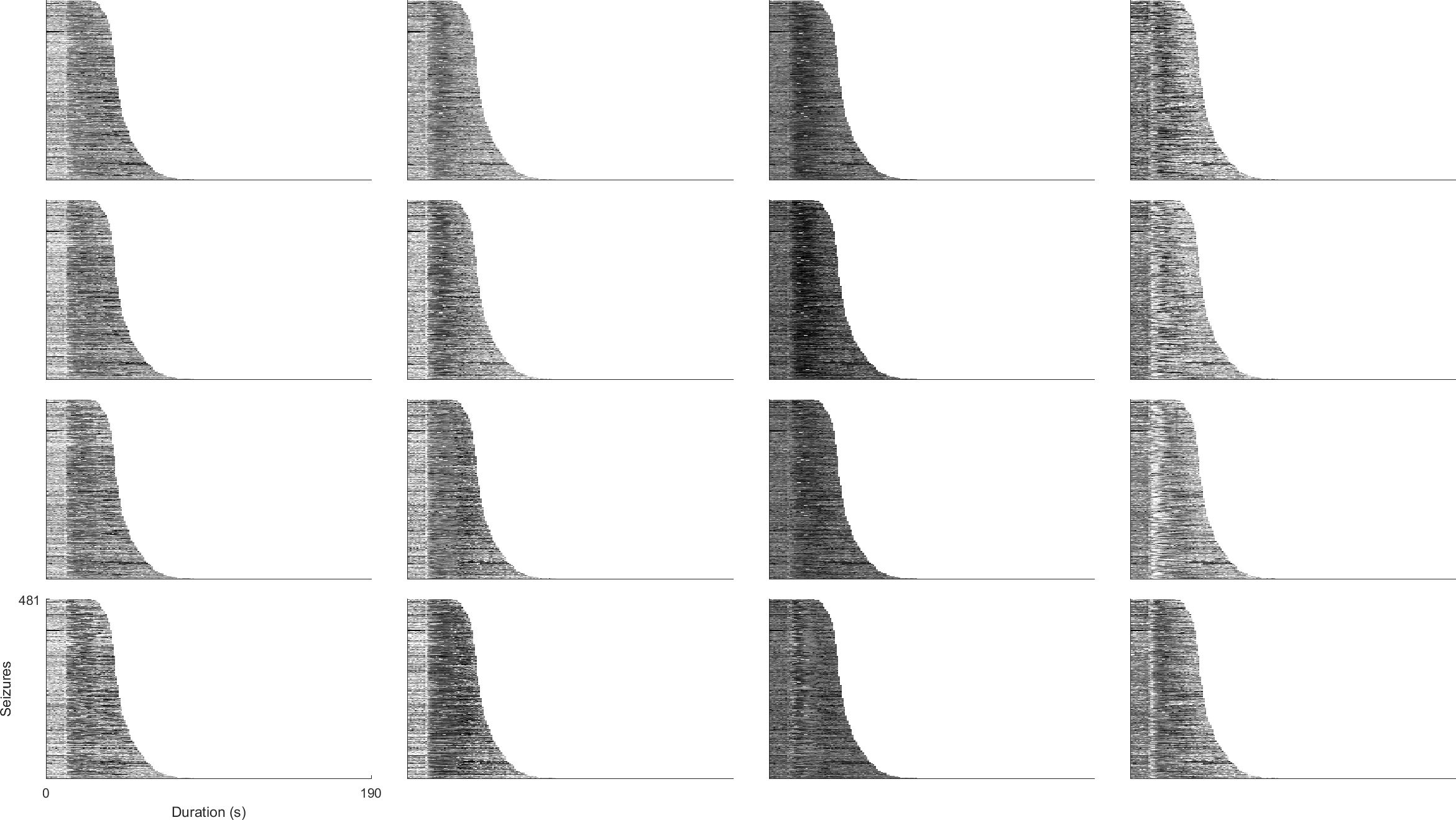

Supplement: S18 Fig — Signal energy during evolution of seizures, sorted by duration, from 10s before seizure onset (marked by arrowhead) to 10s after seizure termination (according to clinicians’ marking). Energy was computed for a 1s sliding window (50% overlap). (TIF) [file pcbi.1006403.s019.tif]

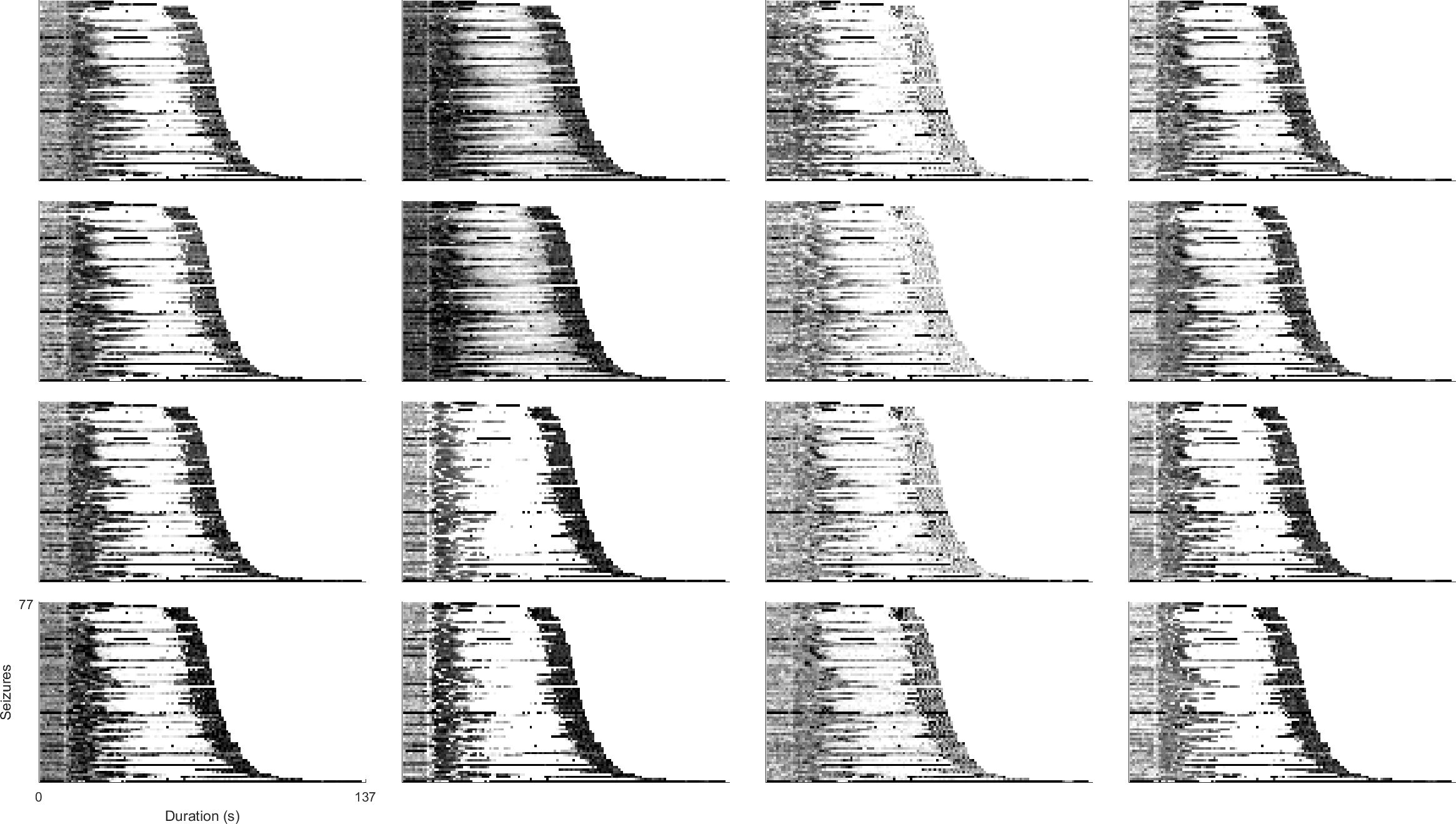

Supplement: S19 Fig — Signal energy during evolution of seizures, sorted by duration, from 10s before seizure onset (marked by arrowhead) to 10s after seizure termination (according to clinicians’ marking). Energy was computed for a 1s sliding window (50% overlap). (TIF) [file pcbi.1006403.s020.tif]
